# Genome wide re-sequencing of newly developed Rice Lines from common wild rice (*Oryza rufipogon* Griff.) for the identification of NBS-LRR genes

**DOI:** 10.1371/journal.pone.0180662

**Published:** 2017-07-11

**Authors:** Wen Liu, Fozia Ghouri, Hang Yu, Xiang Li, Shuhong Yu, Muhammad Qasim Shahid, Xiangdong Liu

**Affiliations:** 1 State Key Laboratory for Conservation and Utilization of Subtropical Agro-bioresources, South China Agricultural University, Guangzhou, China; 2 Department of Tropical Crops, Guangdong Agriculture Industry Business Polytechnic College, Guangzhou, China; Xiamen University, CHINA

## Abstract

Common wild rice (*Oryza rufipogon* Griff.) is an important germplasm for rice breeding, which contains many resistance genes. Re-sequencing provides an unprecedented opportunity to explore the abundant useful genes at whole genome level. Here, we identified the nucleotide-binding site leucine-rich repeat (NBS-LRR) encoding genes by re-sequencing of two wild rice lines (i.e. Huaye 1 and Huaye 2) that were developed from common wild rice. We obtained 128 to 147 million reads with approximately 32.5-fold coverage depth, and uniquely covered more than 89.6% (> = 1 fold) of reference genomes. Two wild rice lines showed high SNP (single-nucleotide polymorphisms) variation rate in 12 chromosomes against the reference genomes of Nipponbare (*japonica* cultivar) and 93–11 (*indica* cultivar). InDels (insertion/deletion polymorphisms) count-length distribution exhibited normal distribution in the two lines, and most of the InDels were ranged from -5 to 5 bp. With reference to the Nipponbare genome sequence, we detected a total of 1,209,308 SNPs, 161,117 InDels and 4,192 SVs (structural variations) in Huaye 1, and 1,387,959 SNPs, 180,226 InDels and 5,305 SVs in Huaye 2. A total of 44.9% and 46.9% genes exhibited sequence variations in two wild rice lines compared to the Nipponbare and 93–11 reference genomes, respectively. Analysis of NBS-LRR mutant candidate genes showed that they were mainly distributed on chromosome 11, and NBS domain was more conserved than LRR domain in both wild rice lines. NBS genes depicted higher levels of genetic diversity in Huaye 1 than that found in Huaye 2. Furthermore, protein-protein interaction analysis showed that NBS genes mostly interacted with the cytochrome C protein (Os05g0420600, Os01g0885000 and BGIOSGA038922), while some NBS genes interacted with heat shock protein, DNA-binding activity, Phosphoinositide 3-kinase and a coiled coil region. We explored abundant NBS-LRR encoding genes in two common wild rice lines through genome wide re-sequencing, which proved to be a useful tool to exploit elite NBS-LRR genes in wild rice. The data here provide a foundation for future work aimed at dissecting the genetic basis of disease resistance in rice, and the two wild rice lines will be useful germplasm for the molecular improvement of cultivated rice.

## Introduction

Common wild rice (*Oryza rufipogon* Griff.), the progenitor of Asian cultivated rice (*Oryza sativa* L.), is widely distributed in the tropics and subtropics of Asia, Papua New Guinea, and Australia [1,2]. Common wild rice has abundant genetic diversity and various resistance genes for the improvement of cultivated rice [3,4]. To fulfill the demands of food supply, there is a need to enhance the crop productivity significantly by exploitation and utilization of genetic resources, particularly those in the gene pool of wild species [4]. However, the natural habitats of wild rice germplasm are becoming sparser due to the activity of modern agriculture, and many wild rice populations have become extinct [5]. As genetic diversity among commercial cultivars has declined, it is difficult to find new resistance genes from existing cultivars for the further improvement of rice. Therefore, sufficient resistance could be managed by exploring wild species [6].

The high-quality map-based sequences of *japonica* cv. Nipponbare and *indica* cv. 93–11 is available at several platforms for rice functional genomics studies, which were used as reference genomes in the previous studies [7]. The next generation sequencing (NGS) technologies have enabled more efficient genome re-sequencing of a large number of genomes at a significantly lower cost than ever before [8]. Although this approach requires resequencing and bioinformatics tools, millions of DNA polymorphisms such as single nucleotide polymorphisms (SNPs), insertions-deletions (InDels) and structural variations (SV) can be obtained by NGS. NGS has provided genome-wide genetic variations in a highly efficient way, and thousands of rice genes have been screened to characterize the biological functions [1,9,10]. NGS play an important role in exploration of gene variations, and it is also considered a favorable toolkit [11,12].

SNPs are the most frequent type of DNA variations in the genomes of most species. The extremely large volume of SNPs makes whole-genome genotyping studies possible in higher plants. InDels and SNPs are of growing importance as molecular markers for crop breeding and improvement programs. InDel markers are relatively cheap and need comparatively simple genotyping with low technical requirements, making them feasible alternatives to laboratories with limited resources [13,14].

Nucleotide-binding site leucine-rich repeat (NBS-LRR) encoding genes form a large polymorphic family in plants, and are divided into nucleotide binding site (NBS) and 1eucine-rich repeat (LRR). NBS-LRR encoding genes play important roles in disease resistance, especially in rice blast and bacterial blight disease [15,16]. Due to the emergence of new physiological races of pathogens, the resistance genes often become ineffective after a few years [17,18]. Therefore, exploring the new resistance genes will gain better knowledge of the disease resistance in rice. Generally, common wild rice withstands in natural environment for long time, and conserved abundant genes, which is beneficial for modern rice breeding [19,20].

China has a rich collection of diverse wild rice populations, and most of them are present in Hainan, Guangdong, Guangxi, Yunnan, Fujian, Taiwan, Hunan, and Jiangxi provinces [4,21,22]. Dongxiang, which is located at 28°14′ N latitude and 116°30′ E longitude, Jiangxi province, China, is considered to be the northernmost region in China and in the world where wild rice is present [9,23]. Dongxiang wild rice is a vast reservoir of beneficial genes that can use to breed cultivated rice, such as broad-spectrum NBS-LRR type resistance genes [9,23].

In the previous studies, the NBS-LRR genes were explored according to the unique primers of conserved sites in homologous sequence, namely resistance gene analogs (RGA) clone [16,18,24]. RGA clone can identify the new genes based on genome database, but we cannot get the full sequences and may lose some novel genes. So far, there are few studies about NBS-LRR encoding genes based on whole genome sequences in common wild rice. In this study, two common wild rice lines, including Huaye 1 and Huaye 2, developed by our research group from a common wild rice indigenous to Dongxiang, Jiangxi province, were re-sequenced through NGS, and mapped onto the reference genomes of Nipponbare and 93–11 to explore NBS-LRR genes. The results may provide elite genes for the breeding of resistant rice.

## Materials and methods

### Plant materials and DNA isolation

Two wild rice lines, i. e. Huaye 1 and Huaye 2, which were developed from one common wild rice indigenous to Dongxiang, Jiangxi province, were re-sequenced through NGS. Genomic DNA was isolated from the leaves of 2-weeks-old seedlings using the modified CTAB (Cetyltrimethyl Ammonium Bromide) method [25].

### Genomic sequences

Following quality assessment, genomic DNA was randomly fragmented by sonication, and the DNA fragments were gel purified and ligated to adapters. Genome DNA was re-sequenced using Illumina (HiSeq 2000) sequencing by the Millennium Genomics -Shenzhen (Shenzhen, Guangdong, China).

The reference genome data of Nipponbare and 93–11 was downloaded from the Rice Information System (Nipponbare: http://plants.ensembl.org/Oryza_sativa/Info/Index. 93–11: http://plants.ensembl.org/Oryza_indica/Info/Index). Using the DNA sequences of the reference genomes as template, the short reads of Huaye 1 and Huaye 2 were aligned to the Nipponbare and 93–11 reference genomes as four groups sequence data (i.e., Huaye 1 and 93–11, Huaye 2 and 93–11, Huaye 1 and Nipponbare, and Huaye 2 and Nipponbare). The adapter sequences or low quality reads (base quality value less than 20) were filtered out using CASAVA v1.8.2 and FastQC software. After filtration, clean data were aligned to the Nipponbare and 93–11 reference genomes by Burrows-Wheeler Aligner (BWA) software, and the redundant reads were filtered out by Picard software.

### Detection of SNPs, InDels and SVs, and GO analysis

Detection of single nucleotide polymorphisms (SNPs) and insertions and deletions (InDels) were performed using Samtools (Command line arguments: samtools mpileup -ugf reference.fa sample.sorted.rmdup.bam | bcftools view -bvcg -> sample.bcf). To eliminate the false positive variations and to identify reliable SNPs and InDels, the variations with the read depths ranged from 5X to 250X were retained by using a commend line (i.e. bcftools view sample.bcf | vcfutils.pl varFilter–d 5 -D 250 > sample.vcf). SNPs and InDels were annotated by using SnpEff software (Command line arguments: SnpEff -no-upstream -no-downstream -s 228 ASM465v1.26 288_filtered.vcf). The distributions of SNPs and InDels were detected along each genome by a sliding window method (distribution of genome 100 kb). We identified SNPs and InDels in different genomic regions, such as introns, untranslated regions (UTRs), coding sequences (CDS) and intergenic regions. The SNPs were differentiated as transition (C/T and G/A) and transversion (C/G, T/A, A/C and G/T) SNPs. Structural variations (SVs) and numbers were detected by BreakDancer (default parameters) software. The densities of SNPs, InDels and SVs were drawn online by Circos (http://circos.ca/). Gene Ontology (GO) enrichment analysis was done using online tool (http://www.geneontology.org) with the “*Oryza sativa*” set as a species background (Nipponbare and 93–11 reference genomes).

### Identification of variations in NBS-LRR genes, their locations and predicted protein-protein interactions

Depending on the SNPs and InDels data variations, GO Slimmer (http://tools.bioso.org/cgi-bin/amigo/slimmer) was used to examine the associations between resistance genes and functional annotation clusters. The online tool Panther Classification System (http://www.pantherdb.org/geneListAnalysis.do) was used for Pathway analysis [26]. The clusters of NBS region were constructed by MEGA 6.0, and the conserved domains of NBS-LRR encoding genes were identified using Pfam platform (http://pfam.sanger.ac.uk/).

The locations of NBS-LRR encoding genes on the chromosomes were drawn by MapDraw V2.1 software [27]. For calculating the gene cluster distributions on 12 chromosomes of rice, NBS-LRR genes with sequence variations were identified according to the distance between two adjacent genes (i.e. less than 200kb) [28,29]. ClustalW was used for multi-sequence alignment of the NBS sequences, and the phylogenetic trees of NBS regions were constructed based on the Bootstrap neighbor-joining (NJ) method by MEGA version 6.0 software [30]. Predicted protein-protein interactions of NBS genes were analyzed using STRING online database (http://www.string-db.org/).

### Experimental validation of NBS conserved domains

For the validation of NBS conserved domains, the primers of NBS sequence were designed using Primer3 [31], with a product length of approximately 700 bp (S1 Table). Polymerase chain reaction (PCR) was used to amplify NBS sequences in a 20 μl volume containing 30 ng template, 0.15 μmol/L primer pairs, 1.0 μl dNTPs (2.0 mmol/l each), one unit Taq polymerase, and 1×PCR buffer (50 mmol/L KCl, 10 mmol/L Tris-HCl pH 8.3, 1.5 mmol/L MgCl_2_, 0.01% glutin). The PCR profile was 94°C for 5 minutes followed by 30 cycles of 94°C for 45 s, 55°C for 45 s, and 72°C for 50 s, and a final extension at 72°C for 5 minutes. PCR products were separated by electrophoresis on a 1.5% agarose gel. A DNA ladder of 100 to 2000 bp was used to estimate the size of the PCR products. Ultimately, PCR products were sequenced and validated by BLAST online tool in NCBI (http://www.ncbi.nlm.nih.gov/).

## Results

### The breeding procedure of wild rice lines

**Fig 1:**
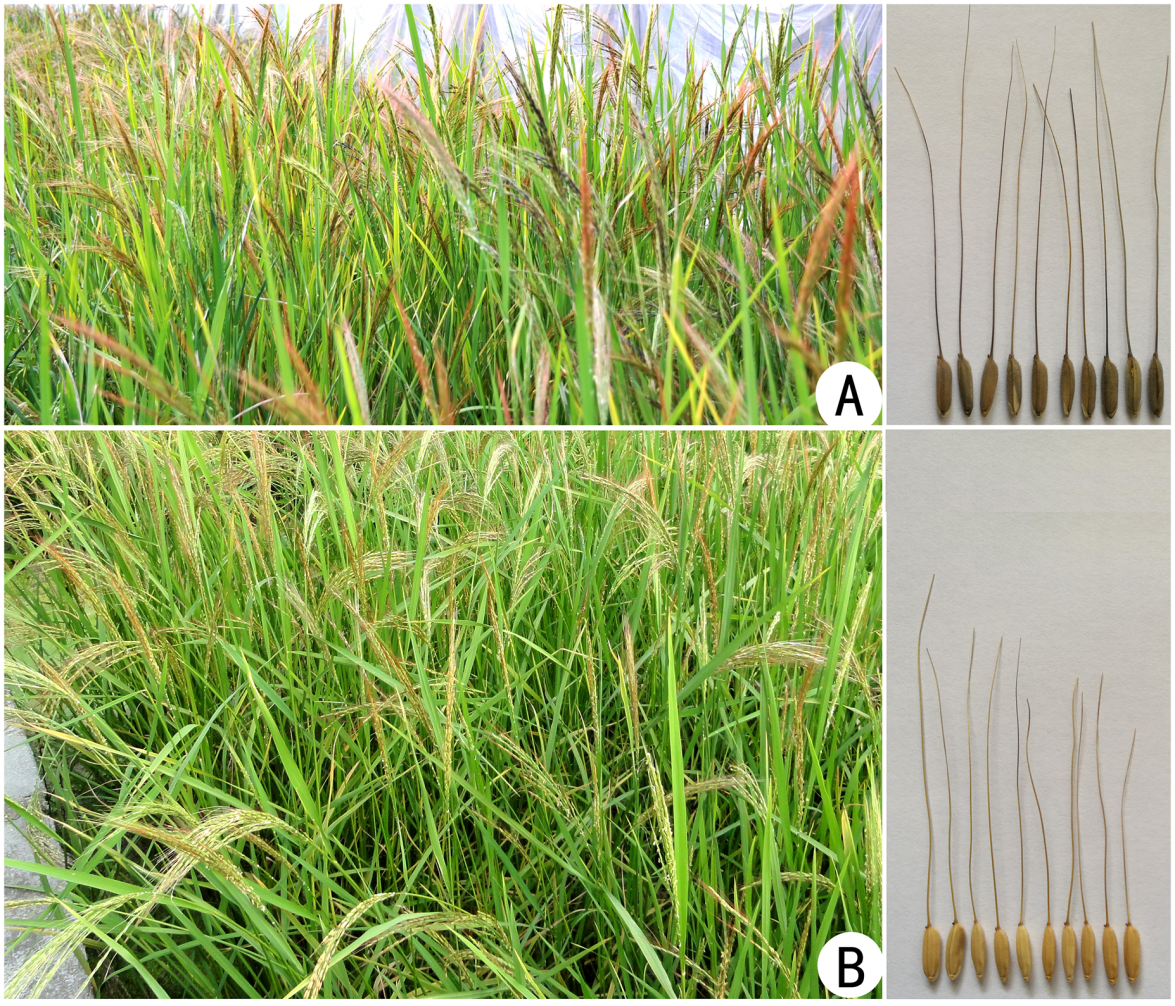
Common wild rice lines at reproductive stage in the field. (A) Huaye 1. (B) Huaye 2. Right panels are representing grains of respective lines. These lines were developed from non-shattering plant with high seed setting of common wild rice.

In this study, one plant with a medium plant structure and non-shattering grains was found in the self-crossed progeny of common wild rice (DXW102) indigenous to Dongxiang, Jiangxi province in 2010. Two plants with more than 80% seed setting were selected from the next generation of non-shattering plant in 2011. These two plants were continuously self-crossed for six generations to attain stability. Subsequently, we developed two stable lines, which were named as “Huaye 1” and “Huaye 2” in 2014 (Fig 1).

### Mapping of Illumina reads

The whole genomes of Huaye 1 and Huaye 2 were mapped onto the 93–11 and Nipponbare reference genomes, and we obtained 12 and 14 G bases high quality clean data, respectively. A total of 128 to 147 million mapped reads (short reads) were generated by BWA software, and the uniquely mapped reads covered more than 87.62% of the sequencing reads across both 93–11 and Nipponbare reference genomes (Table 1). The sequencing reads were distributed evenly on all the rice chromosomes (Figs A-D in S1 File). More than 89% of the mapped sites (> = 1 fold) covered across reference genome, and average folds were between 32.48 to 38.23-fold (Table 1). These results suggested that the sequencing quality of the clean reads was generally high.

**Table 1:** Summary of the genome wide resequencing data of wild rice lines mapped onto the reference genomes.

Reference genome	Sample	Ref. genome length (Bases)	Mapped sites (> = 1 fold)	Coverage (> = 1 fold)	Total reads	Mapped read	Mapped read (%)	Mapped bases (G)	Average fold
93–11*	Huaye 1	373,877,896	334,950,376	89.59	146,292,262	128,182,010	87.62	12.14	32.48
	Huaye 2	373,877,896	337,689,327	90.32	166,563,520	146,723,762	88.09	14.01	37.47
Nipponbare*	Huaye 1	373,245,519	353,150,272	94.62	146,292,262	132,320,977	90.45	12.73	34.09
	Huaye 2	373,245,519	350,432,046	93.89	166,563,520	147,729,706	88.69	14.27	38.23

* http://plants.ensembl.org/Oryza_indica/Info/Index (93–11)

http://plants.ensembl.org/Oryza_sativa/Info/Index (Nipponbare)

### Detection and distribution of variations

We identified genetic variations between the two wild rice lines (Huaye 1 and Huaye 2) and reference genomes (Nipponbare and 93–11), and a total of 4,998,437 SNPs, 670,521 InDels and 22,305 SVs (structural variations) were detected between wild rice lines and reference genomes. The overall genome densities of SNPs and InDels were more than 1,063,472 and 148,774 in wild rice lines compared to the Nipponbare or 93–11 reference genomes, respectively (Table 2). Wild rice lines showed high SNP variation rate (between 1.8 to 5.8 in 1 kb) along the chromosomes (Fig E in S1 File). The highest SNP density was detected on chromosome 6 between Huaye 1 and Nipponbare, and on chromosome 9 between Huaye 2 and Nipponbare. Chromosome 9 and chromosome 10 exhibited the highest SNP variation rate between Huaye 1 and 93–11 reference genome, and Huaye 2 and 93–11 reference genome, respectively. Moreover, the InDels count-length distribution exhibited normal distribution in the two wild rice lines, and most of the InDels polymorphisms were -5~5 bp length (Fig F in S1 File). Then, SNPs, InDels and SVs were analyzed by circos per 100Kb. SNPs, InDels and SVs exhibited correlations at some locations on different chromosomes, including 30-35Mb on chromosome 2, 15-18Mb on chromosome 9 and 8-30Mb on chromosome 12 between the genomes of Huaye 1 and Nipponbare, and also showed the highest density for SNPs, InDels and SVs at the same chromosomal locations (Fig G in S1 File). Other three group’s data (i.e. Huaye 1 vs 93–11; Huaye 2 vs 93–11; Huaye 2 vs Nipponbare) also exhibited the similar density patterns for SNPs, InDels and SVs (Figs H-J in S1 File).

**Table 2:** Summary of the whole genome sequence variations between wild rice lines and reference genomes.

Reference genome	Sample	SNP	InDel	SV
93–11*	Huaye 1	1,337,698	180,404	6,319
	Huaye 2	1,063,472	148,774	6,489
Nipponbare*	Huaye 1	1,209,308	161,117	4,192
	Huaye 2	1,387,959	180,226	5,305
Total		4,998,437	670,521	22,305

*http://plants.ensembl.org/Oryza_indica/Info/Index (93–11)

http://plants.ensembl.org/Oryza_sativa/Info/Index (Nipponbare)

There was a great variation in the frequency of SNP bases substitution, including transitions and transversions, between two wild rice lines and reference genomes (Table 3). A/G transitions (256,021) were the highest, while C/G transversions (29,718) were the lowest between Huaye 2 and Nipponbare reference genome. Generally, SNP bases substitution showed the higher transitions than the transversions between purine and pyrimidine. Moreover, 4,192 and 5,305 SVs were detected between Huaye 1 and Nipponbare reference genome, and between Huaye 2 and Nipponbare reference genome, respectively.

**Table 3:** The frequency of SNP bases substitution between wild rice lines and reference genomes.

Sample	Base	93–11 reference genome				Nipponbare reference genome			
		A	C	G	T	A	C	G	T
Huaye 1	A	—	51,853	237,959	59,654	—	46,564	203,260	53,572
	C	55,295	—	36,627	227,188	48,371	—	31,605	220,915
	G	227,618	36,423	—	55,377	220,817	31,596	—	48,437
	T	59,790	237,539	52,375	—	53,574	204,496	46,101	—
Huaye 2	A	—	41,232	187,341	46,372	—	53,349	232,858	59,193
	C	44,710	—	29,937	181,480	55,466	—	36,871	254,857
	G	182,044	29,718	—	44,940	256,021	36,976	—	55,386
	T	46,780	187,690	41,228	—	59,793	234,656	52,533	—

Rows with gray color indicate the reference bases, and columns with gray color indicate the substituted bases. For example, Row 'A' column 'T' indicates how many 'A' bases have been replaced by 'T' bases. Red cell means the higher bases substitution, and the green means the lower bases substitution.

### Gene annotation

**Fig 2:**
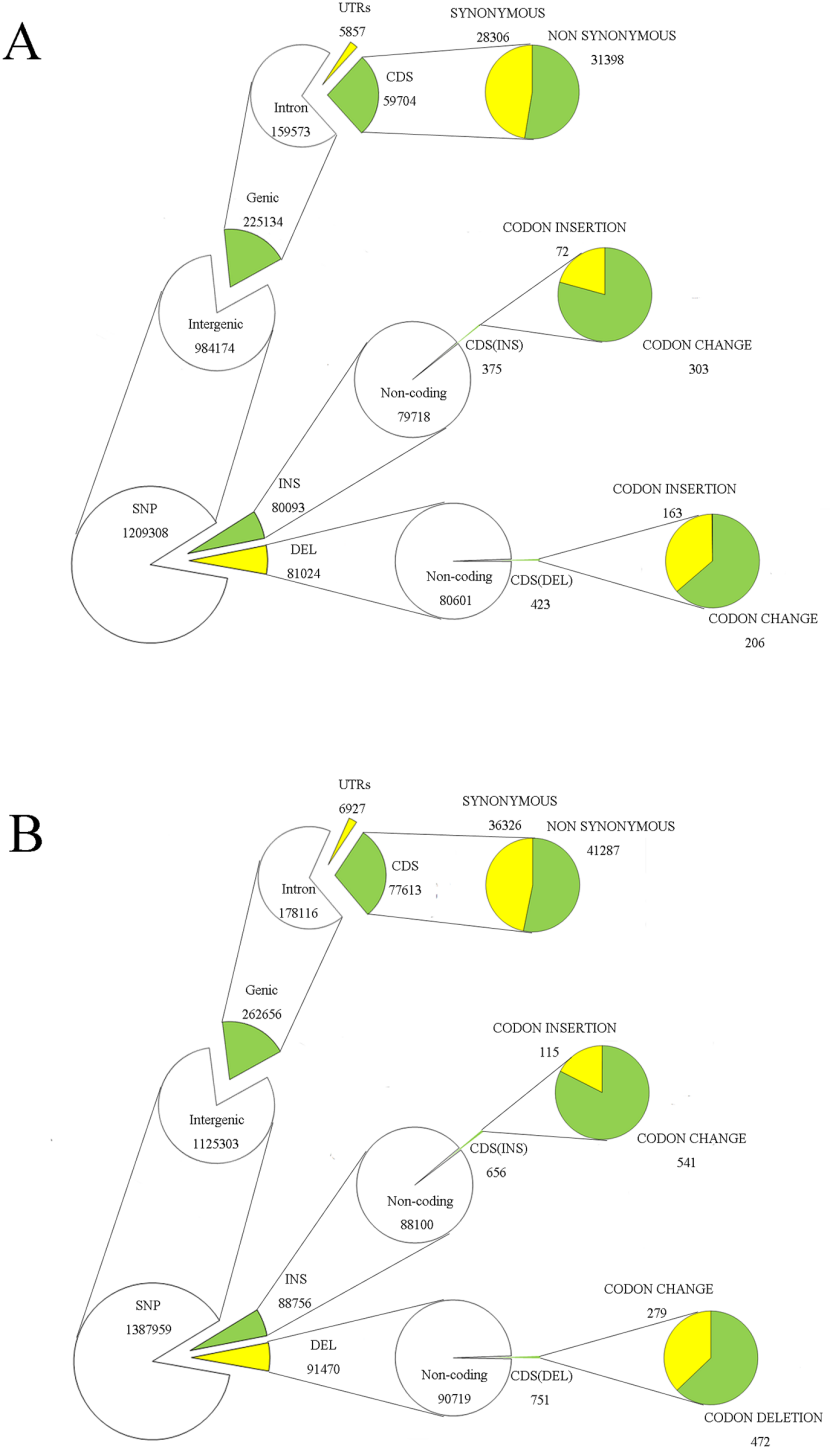
Annotation and distribution of SNPs and InDels between common wild rice lines and Nipponbare reference genome. SNPs, insertions and deletions on the rice pseudomolecules were classified as genic and intergenic regions, and locations within the gene models were annotated. The number of SNPs, insertions and deletions in each class is shown. A and B indicate the distributions of SNPs and InDels in the comparisons of Huaye 1 and Nipponbare reference genome, and Huaye 2 and Nipponbare reference genome, respectively.

The identified candidates were classified into SNPs, insertion (INS) and deletion (DEL) according to their positions in predicted genes in four groups, such as between Huaye 1 and 93–11, between Huaye 2 and 93–11, between Huaye 1 and Nipponbare, and between Huaye 2 and Nipponbare (Fig 2; Fig K in S1 File). The SNP gene annotation showed that most of the SNPs (between 0.8 to 1.2 million) were located in intergenic region, while few SNPs were located in genic region (0.22–0.28 million). Intron, UTRs (untranslated regions) and CDS (coding sequence) were classified in genic region, and there was higher ratio of non-synonymous SNPs than synonymous in the CDS of four groups. We detected higher number of InDels in non-coding sequence than CDS. Overall, the highest SNPs and InDels variations were detected between Huaye 2 and Nipponbare reference genome, while the lowest variations were detected between Huaye 1 and Nipponbare reference genome.

We conducted gene ontology (GO) enrichment analysis with the ‘*Oryza sativa*’ set as a species background for the functional annotation of genes. Genes were classified into three categories, including the cellular component, molecular function and biological process; and mutant genes of cell part, binding and metabolic process were found to be abundant in these three categories. Moreover, enriched GO categories were mainly distributed in the terms of cell part, cell, organelle, binding, catalytic activity, metabolic process, cellular process and single-organism (Figs L-M in S1 File). We detected significant gene variations between two wild rice lines, and a total of 55.1% and 53.1% common genes were detected in two lines compared to the reference genomes of Nipponbare and 93–11, respectively. About 26.1% (2183) and 20.8% (1735) genes depicted sequence variations in Huaye 1 and Huaye 2 when 93–11 was used as a reference genome, while 12.2% (1088) and 32.7% (2911) genes exhibited sequence variations in Huaye 1 and Huaye 2 when Nipponbare was used as a reference genome, respectively (Fig N in S1 File).

### Identification of NBS-LRR genes and their validation

**Fig 3:**
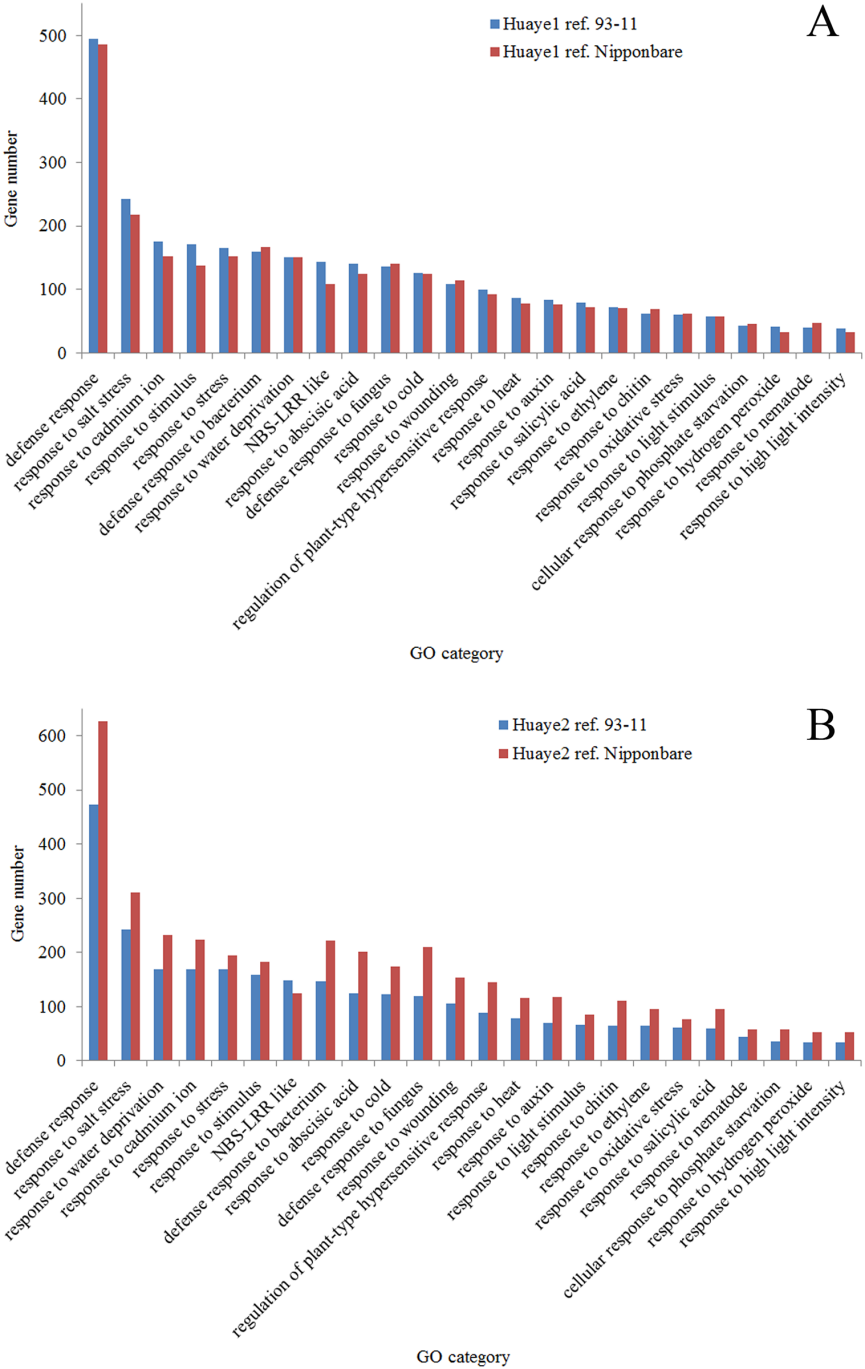
Functional characterization of the resistance genes into gene ontology (GO) terms. (A) and (B) indicate the annotation of resistance genes that were detected in the comparisons of Huaye 1 and reference genomes, and Huaye 2 and reference genomes of Nipponbare and 93–11, respectively.

To further explore the functions of the NBS-LRR candidate genes, we annotated resistance genes by using the gene ontology database and panther classification database. The candidate genes from each group were classified into important gene categories, which play crucial roles during growth and development. Abundant resistance genes were identified in different GO terms, and defense response genes were the highest (more than 480 genes) in two wild rice lines, while the genes related to response to high light intensity (about 50 genes) were the lowest (Fig 3). We detected differences between wild rice lines and reference genomes for NBS-LRR genes, and more than 108 NBS-LRR candidate genes were functionally annotated in each group (i.e. Huaye 1 vs 93–11, Huaye 2 vs 93–11, Huaye 1 vs Nipponbare, and Huaye 2 vs Nipponbare) (Table 4).

**Table 4:** Classification of NBS-LRR genes that showed sequence variations in two wild rice lines.

Sample	Genome	Subfamily			Total
		NBS-LRR	NBS	LRR	
Huaye 1	93–11	9	1	133	143
	Nipponbare	5	1	102	108
Huaye 2	93–11	8	1	139	148
	Nipponbare	4	1	119	124

**Fig 4:**
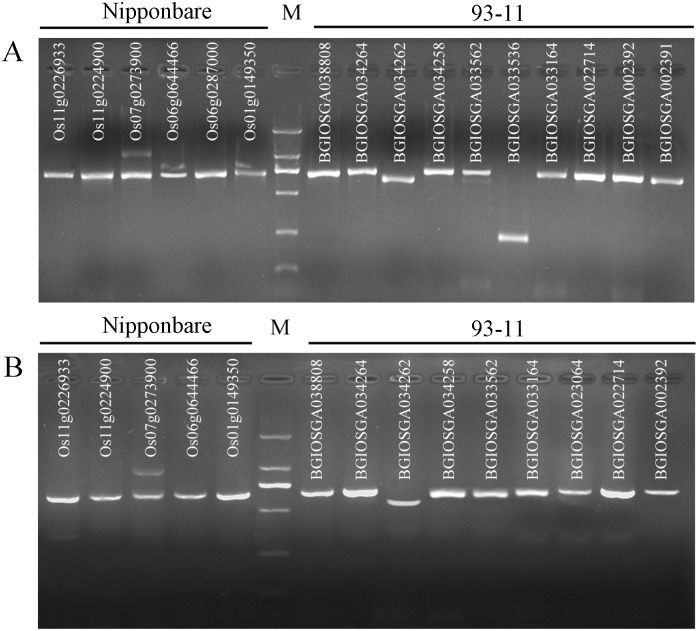
Validation of NBS like genes in wild rice genome. (A) and (B) indicate the PCR products of Huaye 1 and Huaye 2 mapped onto the reference genomes, respectively. M represents DNA Marker, and the vertical letters indicate the gene ID.

To validate the NBS-LRR encoding genes, which were identified by rice genome sequencing, including both or either NBS or LRR region, we selected the NBS regions by gene annotation in Nipponbare and 93–11 reference sequences. Then we designed the primers based on the partial sequences (approximately 700 bp) of selected NBS regions and amplified the target sequences by PCR. The PCR results showed that 16 and 14 primer pairs from Huaye 1 and Huaye 2 could simultaneously generate approximately 700 bp PCR products (BGIOSGA033536 in Huaye 1 generated almost 230 bp) from the genomic DNA of both Nipponbare and 93–11 reference genomes, which revealed 100% accuracy of NBS domain (Fig 4). Finally, PCR products were sequenced and validated by BLAST online tool, and the products were mostly associated with NBS-LRR genes, while some products were associated with other resistance or *Pi* genes, and the sequences identity were ranged from 93% to 100% (S2 Table).

### The distribution of NBS-LRR genes in rice genome

**Fig 5:**
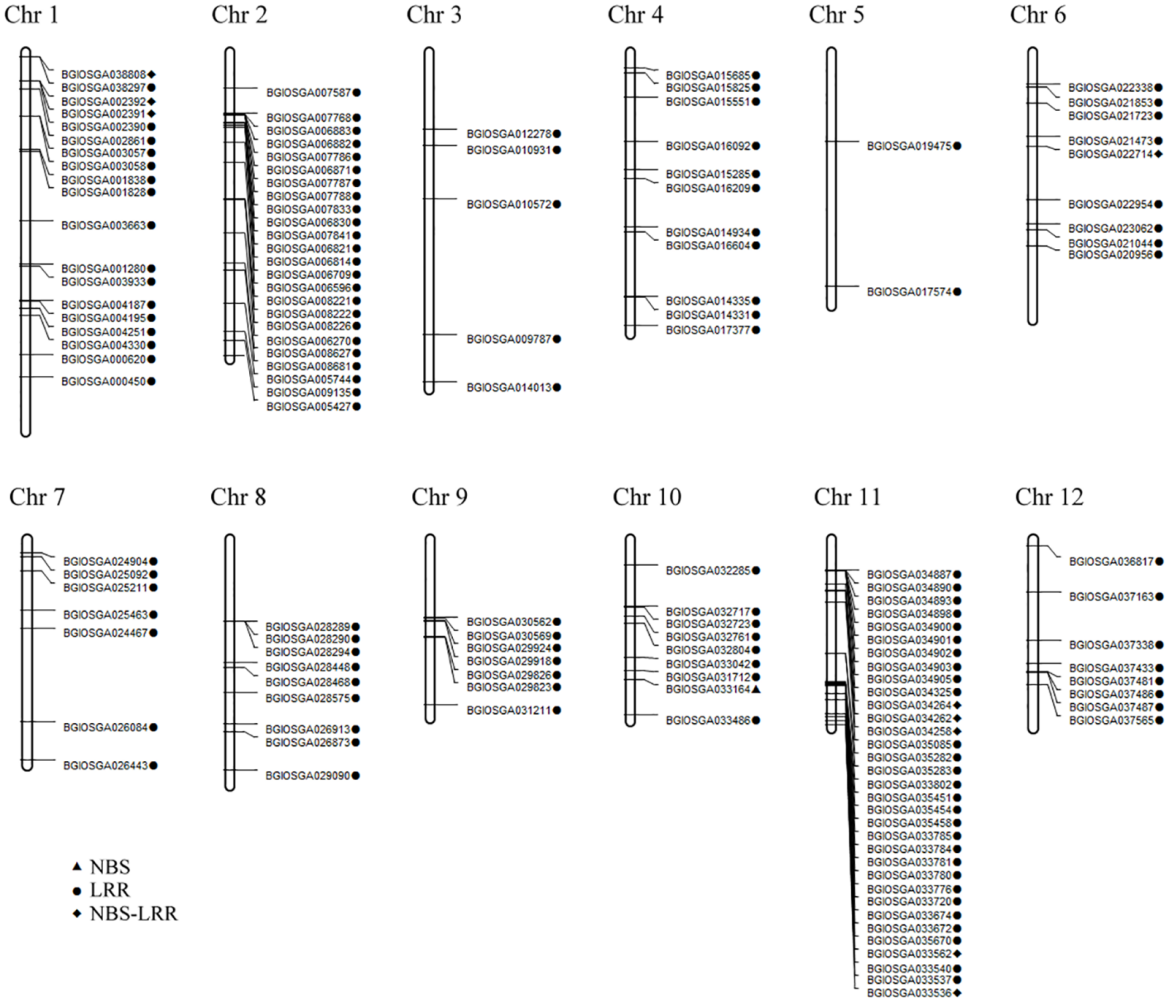
Density map of NBS-LRR resistance genes that were detected between Huaye 1 and reference genome of 93–11.

NBS-LRR genes were distributed over all the rice chromosomes, and the number of LRR regions were significantly higher than NBS regions in four density maps (i.e. Huaye 1 vs Nipponbare, Huaye 1 vs 93–11, Huaye 2 vs Nipponbare, and Huaye 2 vs 93–11), and genes were unevenly distributed across the 12 chromosomes (Fig 5; Figs O-Q in S1 File). A total of 17 and 33 genes were detected on chromosome 11 by aligning Huaye 1 to the Nipponbare and 93–11 reference genomes, respectively. The highest number of genes (37) was found on chromosome 11 between Huaye 2 and 93–11 reference genome, while 16 genes were detected on chromosomes 2 and 11 between Huaye 2 and Nipponbare reference genome (S3 Table).

We then compared the gene clusters of two wild rice lines with the reference genomes, and less than 200kb distance between two adjacent genes were considered as a gene cluster [29,30]. Abundant NBS-LRR gene clusters were detected in both wild rice lines. A total of 21 and 22 gene clusters were found in Huaye 1, and 23 and 27 gene clusters were detected in Huaye 2 by mapping onto the references genomes of 93–11 and Nipponbare, respectively. Chromosome 11 had the most number of gene clusters between two wild rice lines and 93–11 reference genome, while the most number of gene clusters were found at chromosome 4 between wild rice lines and Nipponbare reference genome (S3 Table). Overall, chromosomes 11 and 4 of two wild rice lines exhibited the highest number of NBS-LRR encoding genes and gene clusters by comparing with the reference genomes of Nipponbare and 93–11.

### Clustering and protein-protein interaction at NBS regions

**Fig 6:**
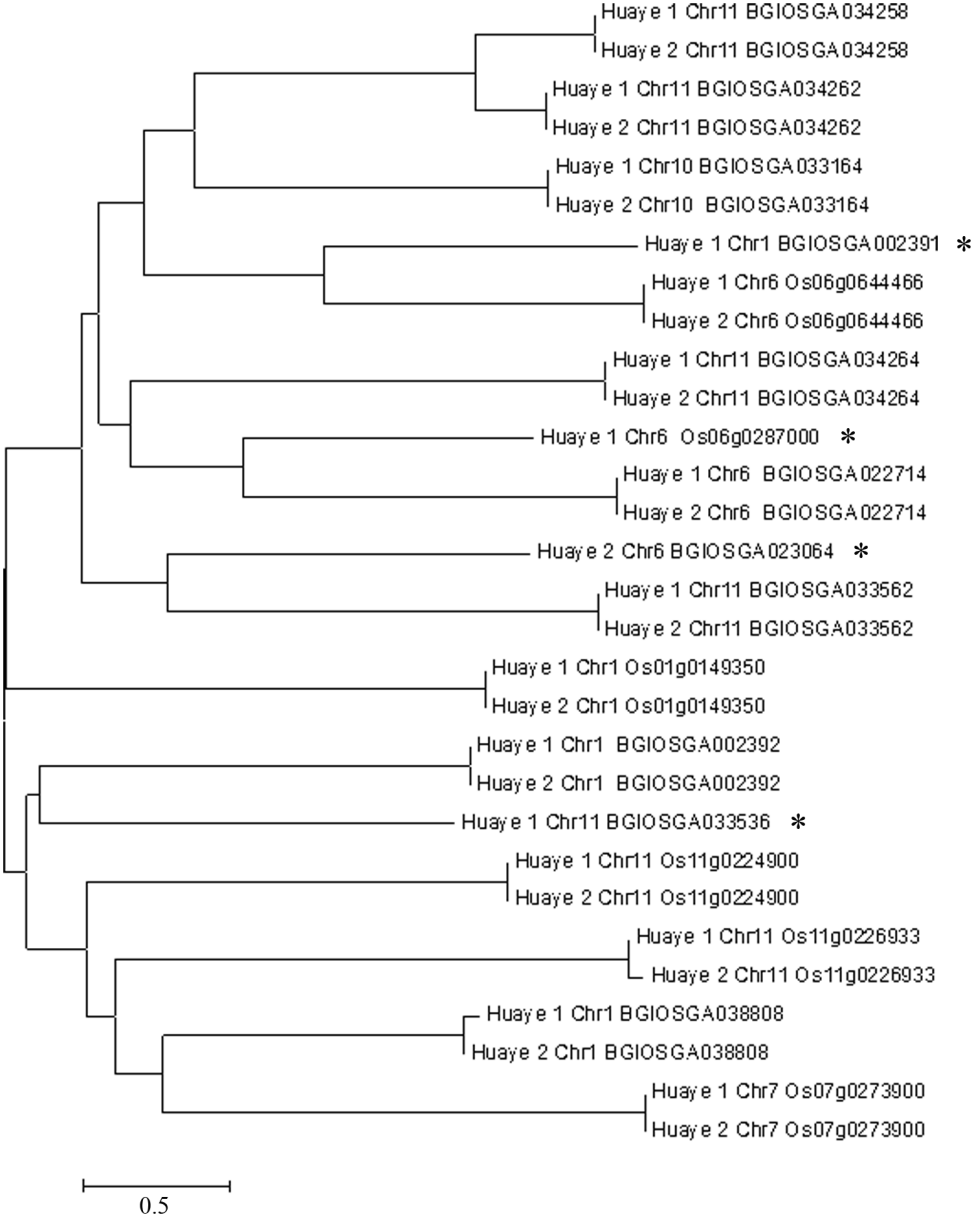
Phylogenetic relationships between NBS regions in two wild rice lines. The names in each branch represent wild rice lines, chromosome number and gene ID. *indicates the novel NBS like genes that were explored from particular reference genome. The scale bar indicates the genetic distance.

NBS regions were structurally conserved domains in NBS-LRR genes. In total, 16 and 14 variable NBS sequences, from Huaye 1 and Huaye 2, were selected for genetic analysis. A total of 30 NBS regions were amplified and sequenced. Phylogenetic tree was used to unambiguously identify the allelic relationship for each gene. In this study, 30 NBS regions were divided into two groups, and the genetic relationship of same gene ID showed the similar genetic distance between two different wild rice lines. However, four genes (Os06g0287000, BGIOSGA002391, BGIOSGA023064, and BGIOSGA033536) revealed independent branches in phylogenetic trees (Fig 6).

Tajima’s neutrality test of NBS regions was done by using MEGA version 6.0 software. In our study, 16 and 14 NBS sequences from Huaye 1 and Huaye 2 were compared for segregating sites, nucleotide diversity and Tajima’s D test. The segregating sites of Huaye 2 (1825) were appreciably higher than Huaye 1 (422), while the nucleotide diversity was very similar in two wild rice lines. Tajima’s D test showed that Huaye 1 (6.440) had higher polymorphisms than Huaye 2 (6.174). Moreover, Tajima’s neutrality test demonstrated that Huaye 1 had the higher NBS structural divergence than Huaye 2 (Table 5).

**Table 5:** Tajima's neutrality test on the NBS sequence data of two wild rice lines.

Sample	Number of sequences	Number of segregating sites	Nucleotide diversity	Tajima’s D test statistic
Huaye 1	16	422	0.745	6.440
Huaye 2	14	1825	0.742	6.174

**Fig 7:**
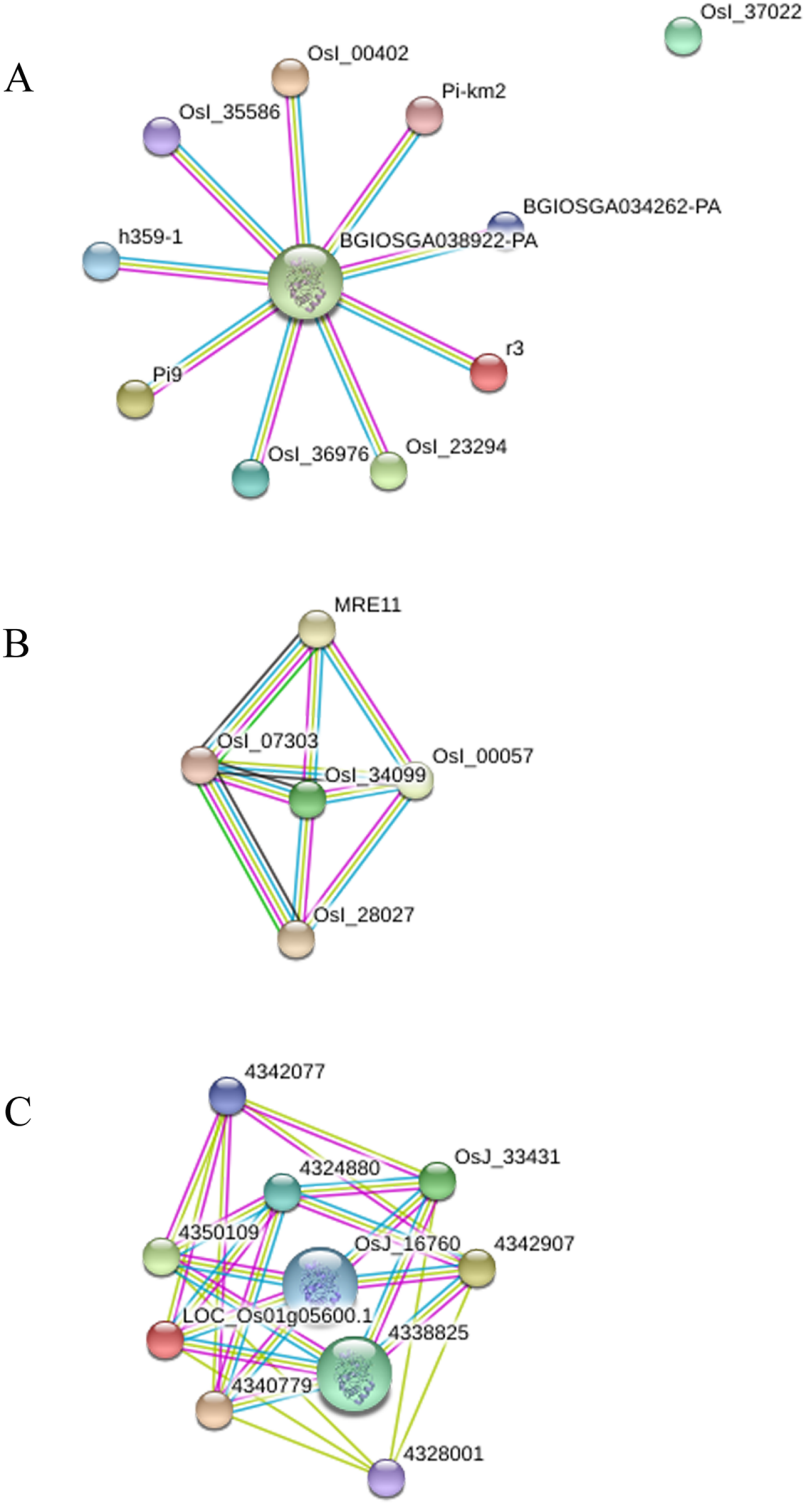
Protein-protein interaction network of NBS-related genes associated with the resistant factors. (A) and (B) indicate the protein-protein interaction network of NBS-related genes between wild rice lines and reference genome of 93–11, (C) indicates the protein-protein interaction network of NBS-related genes between wild rice lines and reference genome of Nipponbare.

NBS genes, which annotated based on Nipponbare and 93–11 reference genomes, were used for protein-protein interaction analysis by online database STRING. All the NBS genes annotated with Nipponbare reference genome displayed interactions with the Cytochrome C protein (Os05g0420600 and Os01g0885000) and a heat shock protein (HSP90; Os06g0716700). We found that most of the NBS genes annotated with 93–11 reference genome also interacted with the Cytochrome C protein (BGIOSGA038922), while some of the NBS genes interacted with DNA-binding activity (BGIOSGA017204, BGIOSGA027555), Phosphoinositide 3-kinase (Pi3-k; BGIOSGA002603) and a coiled coil region (BGIOSGA006432) (Fig 7). All these results suggest that the NBS genes, explored in the present study, interacted with the Cytochrome C protein, heat shock protein, DNA-binding activity, Pi3-k and coiled coil region.

## Discussion

Rice not only has high quality sequences in the public genome but also a model plant system for genomics studies. The release of well-characterized rice genome has been shown to perform well in functional genomics in recent years [8,32]. Next-generation sequencing (NGS) enables the identification of markers such as insertion and deletions (InDels) and single-nucleotide polymorphisms (SNPs) in a simple, efficient, rapid, relatively low cost, and robust manner. SNPs are the most frequent type of DNA variations in the genomes of most species, and are important as DNA markers in crop genetics research, which can predict gene functions. A total of 67,051 SNPs have been identified by comparing a *japonica* rice cultivar “Koshihikari” and Nipponbare reference genome by whole-genome resequencing. Recently, some studies have found many polymorphisms between the reference genome and cultivated rice at different sequencing depths and the results showed that SNPs were less than 100 thousand in each accession [13,33,34]. In this study, over 30-fold sequencing depth and 89% coverage was sufficient to detect DNA polymorphisms, and SNPs were over 10 times (more than 1 million SNPs) in two wild rice lines than cultivated rice [13,33,34]. The InDel polymorphisms could be used as a DNA marker, and the use of InDel polymorphisms may obtain millions of DNA polymorphisms from massive sequence data. Genome Wide Association Study (GWAS) is a low-coverage sequencing approach, but the requirement of low coverage and large sample number limit the application in small populations [1]. Here, two wild rice lines generated significant genetic polymorphisms, which would be an excellent gene resource for rice molecular breeding and improvement. Actually, the two wild rice lines had shown an excellent plant growth and resistant to diseases and insects. We didn’t find any susceptible plants in the two wild lines for six generations during our field investigations, even though we didn’t use any pesticide/insecticide during all growth stages.

SNPs are abundant sequence alterations that affect genic or intergenic regions. We detected lower number of SNPs in genic regions (less than 0.26 million) than intergenic regions (more than 0.82 million) in wild rice, and non-synonymous SNPs were higher than synonymous in CDS. These results are consistent with the previous studies, who also found that most of the high-quality SNPs were located in intergenic regions, and only few were located in CDS regions, and the ratio of non-synonymous SNPs was higher than synonymous in the predicted genes of CDS regions [35,36]. The resequencing of 50 accessions of cultivated and wild rice showed that most of the InDels were located in intergenic regions, and below 1% (8,232) were located in CDS region [37]. Similarly, we found 375 to 751 InDels in CDS region, while most of InDels were detected in non-coding regions.

Nipponbare and 93–11 are classified as *japonica* and *indica* rice, respectively, and selection of a reliable reference genome play a significant role to reveal the polymorphisms accurately [36]. Usually, Nipponbare genome is considered as an excellent template for rice genome re-sequencing. 93–11 is also an important rice germplasm for rice breeding, and the release of 93–11 genome provided a new way to explore the genes [22,38]. In this study, we used two reference genomes to explore NBS-LRR encoding genes, and the results showed significant polymorphisms between wild rice and two reference genomes. Moreover, we detected some peculiar genes between wild rice and reference genomes of Nipponbare and 93–11.

For long term evolution in natural environment, common wild rice conserve abundant resistant genes [3,4], such as the defense response genes, response to physical and physiological stress, also includes abundant new NBS-LRR genes. NBS-LRR genes are the broad-spectrum resistance genes in plants. These genes are expected to be orthologous, as they have common targets for stress response [39]. Except the NGS technologies, the identification of NBS-LRR and other useful genes by PCR using degenerate primers according to sequence similarity is a common method [15,18]. However, the disadvantages of this method are sequence fragmentation and missing of some unexpressed genes. In the cultivated rice genome, more than 400 NBS-LRR genes have been identified [18]. We explored more than four hundred NBS-LRR genes in two wild rice lines developed from common wild rice, and validated the NBS domain by PCR amplification, and the results demonstrated that 100% NBS domains were authentic. Moreover, we investigated the variations among these genes in whole genome, and more than 108 genes exhibited base differences in both wild rice lines (Table 4). The distribution and gene clusters of NBS-LRR encoding genes indicated that wild rice have abundant novel resistant genes, which could be the potential genetic resource for the breeding of cultivated rice. These results revealed that NGS offered fast and accurate mechanisms for the exploration of vital genes.

Although the NBS-LRR genes are believed to participate in defense mechanism [40,41], most of the previous studies were focused on blast and immunity in rice. The protein of pathogen is perceived by R proteins through direct or indirect recognition mechanisms. Direct recognition relies on physical binding of effectors to R proteins, and indirect recognition is based on the perception of effector-induced modifications of host proteins [24,42,43]. However, the functions of most of them, such as protein-protein interaction, are unknown. Protein-protein interaction indicated that most of the NBS genes interacted with the Cytochrome C protein, which is a component of the electron transport chain in mitochondria.

## Conclusion

In this study, we compared the whole genome sequences of two wild rice lines with one another and to the reference genomes of Nipponbare (*japonica* cultivar) and 93–11 (*indica* cultivar). We detected high genetic variations (SNPs, InDels and SVs) at more than 32.48-fold genome coverage depth in wild rice lines. Hundreds of NBS-LRR encoding genes were explored, and chromosome 11 displayed most of the variations in NBS-LRR regions between wild rice lines and reference genomes (cultivated rice). The identification and validation of these NBS domains showed high genetic diversity in wild rice lines, and NBS genes mostly interacted with the cytochrome C protein (Os05g0420600 and Os01g0885000 and BGIOSGA038922). All these polymorphisms will provide gene resources for genetic improvement of cultivated rice through molecular breeding and biotechnology. These two lines will be useful germplasm for breeders for the improvement of rice crop.

## Supporting information

S1 File Genome sequence variations between wild rice lines and reference genomes. Reads distribution of Huaye 1 mapped against the reference genome of Nipponbare and 93-11(Figs A-B). Reads distribution of Huaye 2 mapped against the reference genomes of Nipponbare and 93–11 (Figs C-D). SNP change rate in different chromosomes of wild rice lines (Fig E). The distribution of the length of InDels in two wild rice lines (Fig F). Summary of whole genome variations between wild rice lines and reference genomes (Figs G-J). Annotation and distribution of SNPs and InDels between wild rice lines and 93–11 reference genome (Fig K). Gene ontology (GO) enrichment analysis of mutant genes detected in wild rice lines (Figs L-M). Venn diagram illustrating the proportion of shared gene clusters between wild rice lines and reference genomes (Fig N). Distribution map of NBS-LRR resistance genes that were detected between wild rice lines and reference genomes (Figs O-Q).
(PDF)

S1 Table Information of primers used for the verification of NBS-LRR genes. (DOCX)

S2 Table BLAST validation of NBS-LRR gene sequences by NCBI. (DOCX)

S3 Table The distribution of NBS-LRR genes in rice genome. (DOCX)
