# Supplementary material for: Genome wide re-sequencing of newly developed Rice Lines from common wild rice (Oryza rufipogon Griff.) for the identification of NBS-LRR genes
Source: PLoS One. 2017 Jul 11;12(7):e0180662. doi: 10.1371/journal.pone.0180662 (PMC5507442; doi:10.1371/journal.pone.0180662)
Supplement: S1 File — Reads distribution of Huaye 1 mapped against the reference genome of Nipponbare and 93-11(Figs A-B). Reads distribution of Huaye 2 mapped against the reference genomes of Nipponbare and 93–11 (Figs C-D). SNP change rate in different chromosomes of wild rice lines (Fig E). The distribution of the length of InDels in two wild rice lines (Fig F). Summary of whole genome variations between wild rice lines and reference genomes (Figs G-J). Annotation and distribution of SNPs and InDels between wild rice lines and 93–11 reference genome (Fig K). Gene ontology (GO) enrichment analysis of mutant genes detected in wild rice lines (Figs L-M). Venn diagram illustrating the proportion of shared gene clusters between wild rice lines and reference genomes (Fig N). Distribution map of NBS-LRR resistance genes that were detected between wild rice lines and reference genomes (Figs O-Q). (PDF) [file pone.0180662.s001.pdf]

**Supporting Information**

**S1 File**

**Genome sequence variations between wild rice lines and reference genomes**

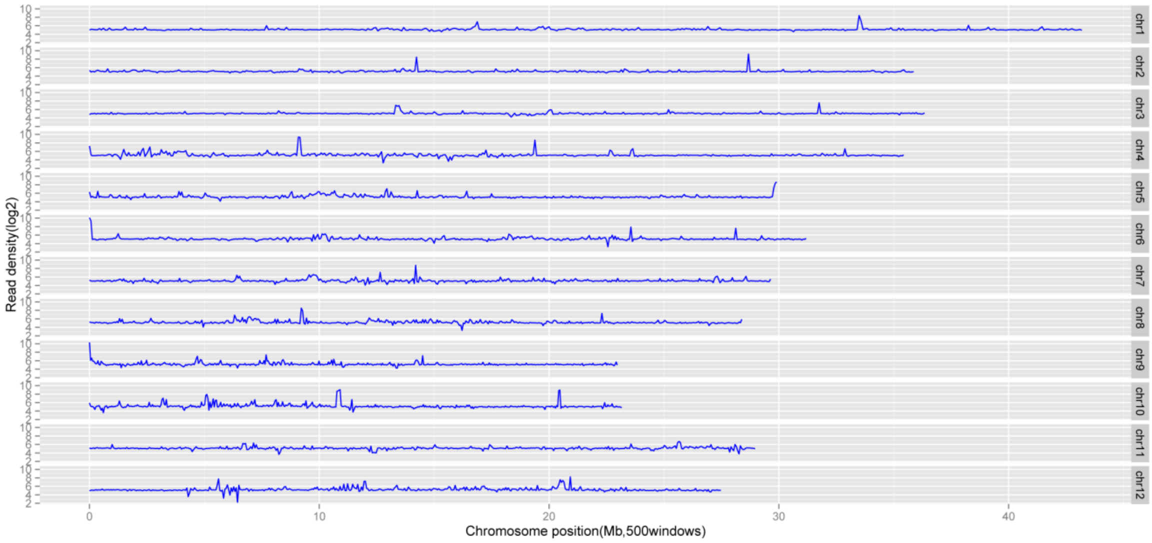


**Figure A. Reads distribution of Huaye 1 mapped against the reference genomes of Nipponbare.**

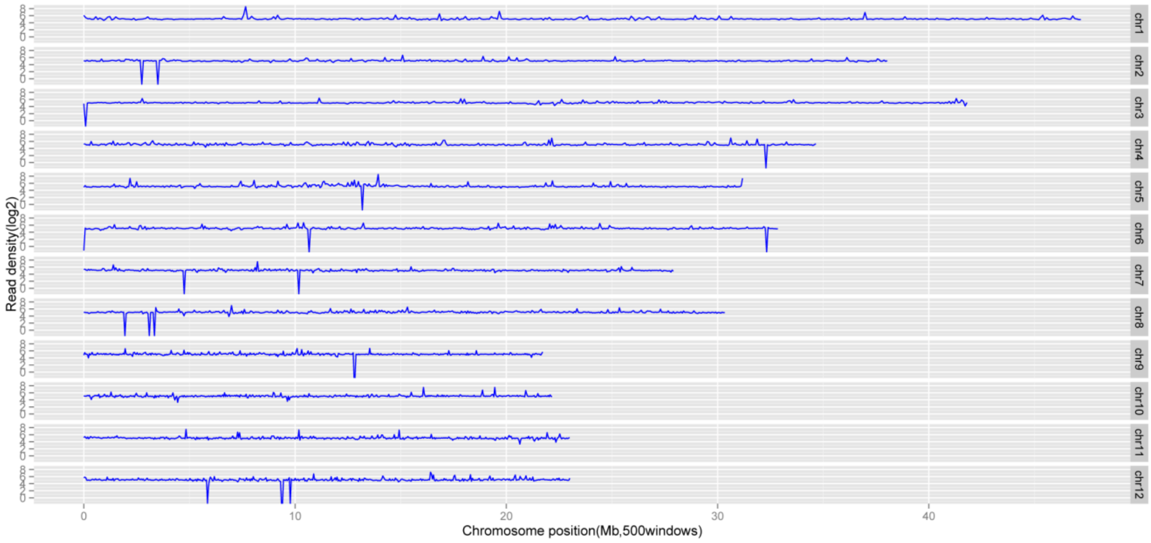


**Figure B. Reads distribution of Huaye 1 mapped against the reference genomes of 93-11.**

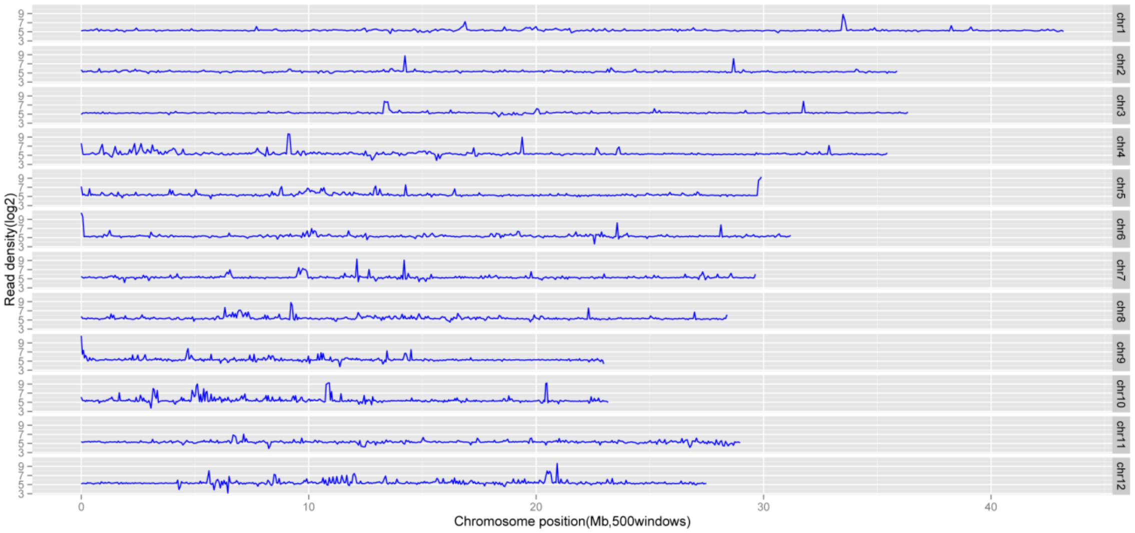


**Figure C. Reads distribution of Huaye 2 mapped against the reference genomes of Nipponbare.**

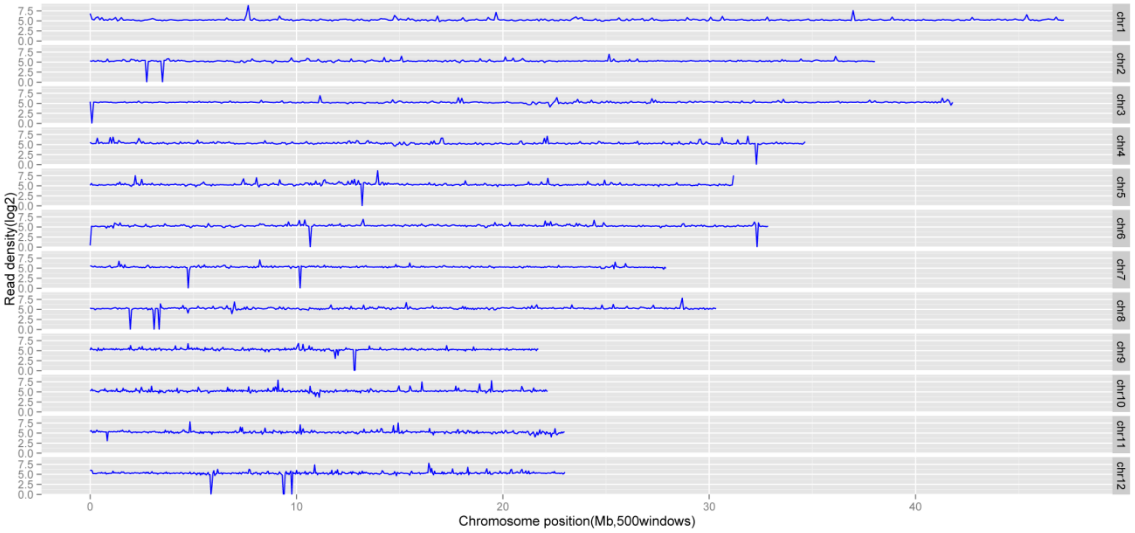


**Figure D. Reads distribution of Huaye 2 mapped against the reference genomes of and 93-11.**

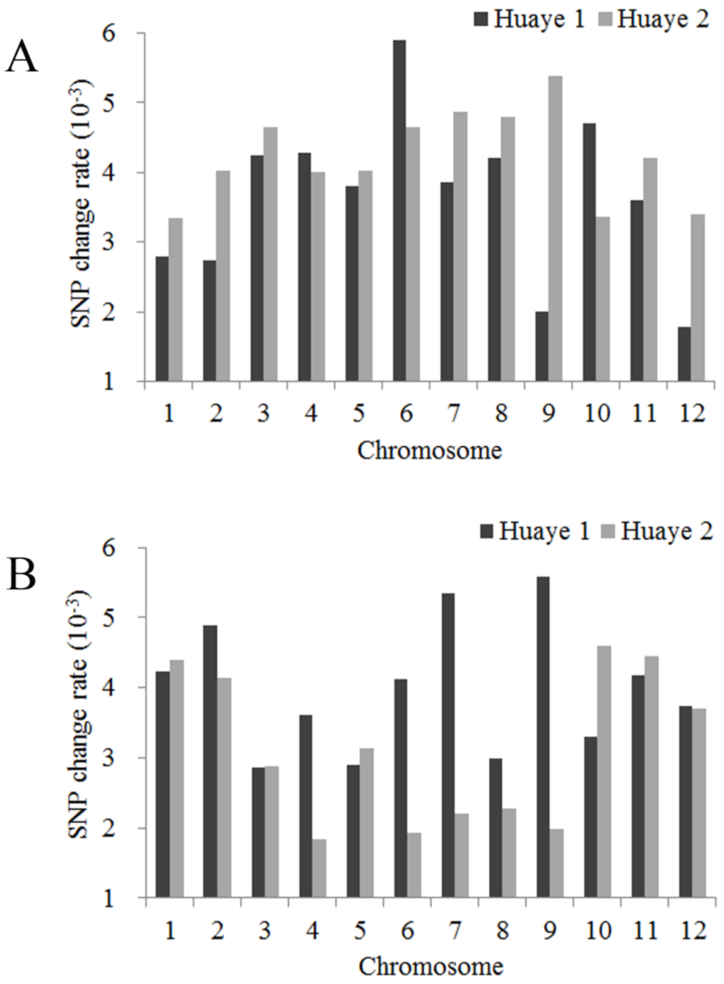


**Figure E. SNP change rate in different chromosomes of wild rice lines.**

(A) SNP change rate between wild rice lines and Nipponbare reference genome. (B) SNP change rate between wild rice lines and 93-11 reference genome.

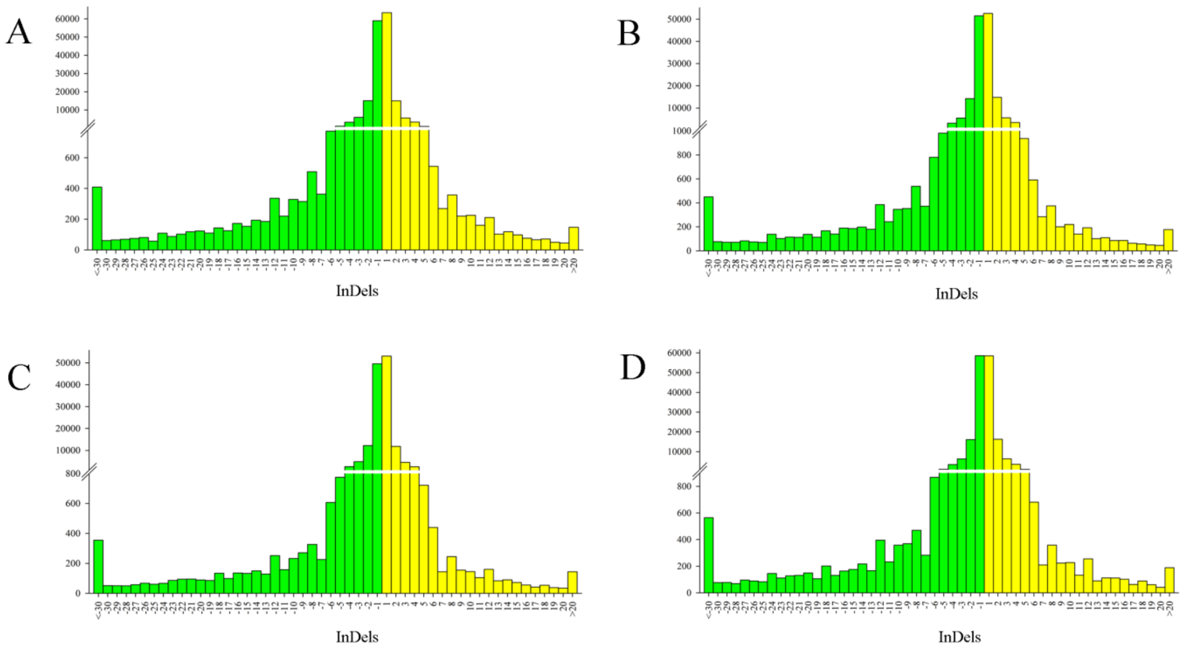


**Figure F. The distribution of the length of InDels in two wild rice lines.**

(A) The distribution of InDels between Huaye 1 and 93-11. (B) The distribution of InDels between Huaye 1 and Nipponbare. (C) The distribution of InDels between Huaye 2 and 93-11. (D) The distribution of InDels between Huaye 2 and Nipponbare. The x-axis represents the number of nucleotides of insertions (yellow) and deletions (green). The y-axis represents the number of InDels at each length.

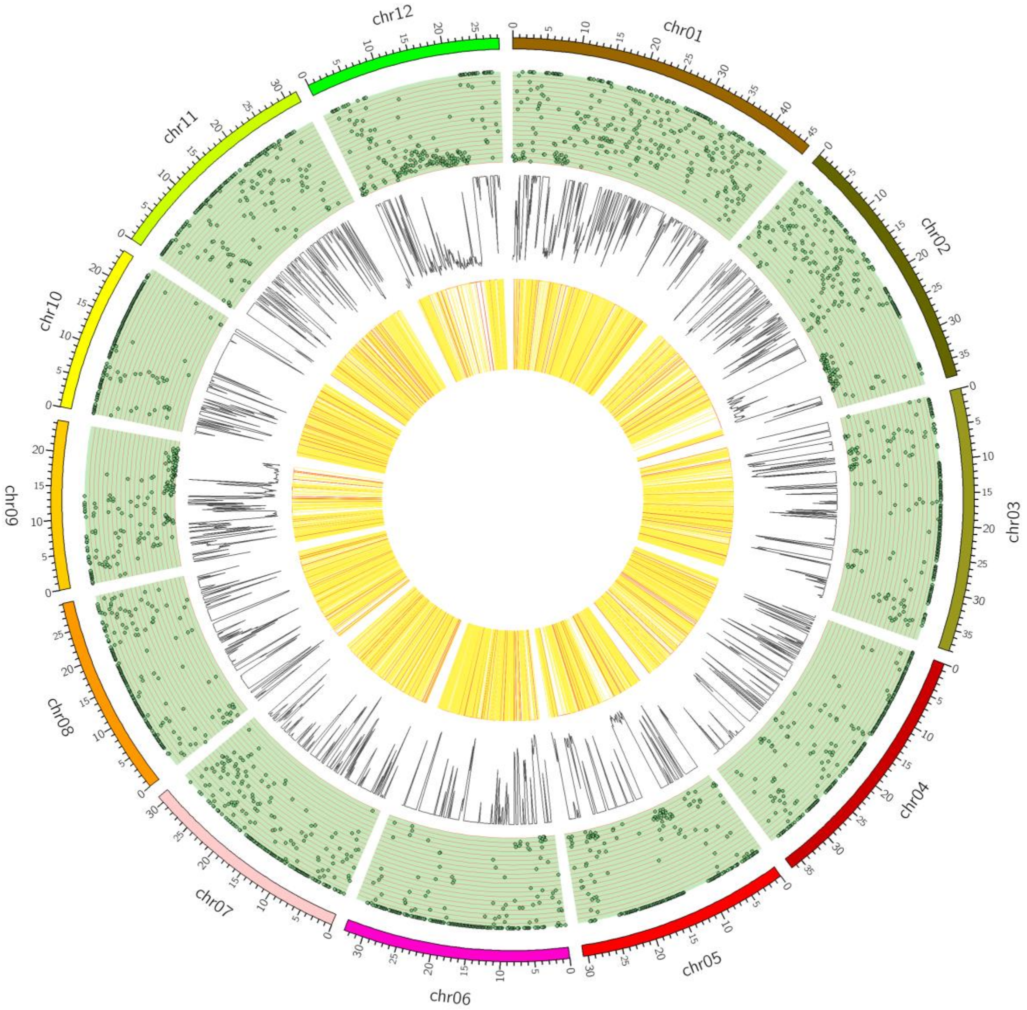


**Figure G. Summary of whole genome variations between Huaye 1 and Nipponbare reference genome.**

Concentric circles show the different features that were drawn using the Circos platform. The 12 chromosomes are presented along the perimeter of each circle. The circles from outside to inside indicate the coordinate of chromosome for SNP density, InDel density and SV density; the distribution unit of genome is 100Kb.

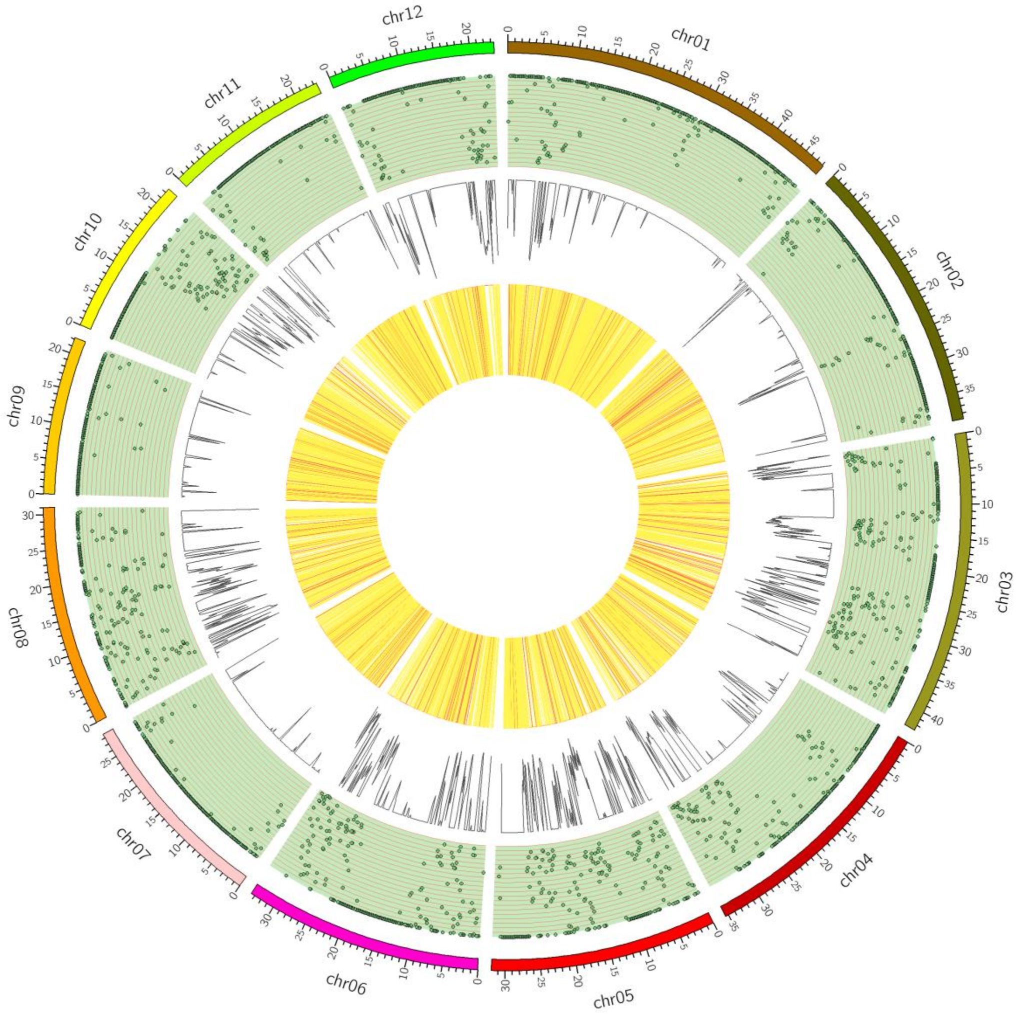


**Figure H. Summary of whole genome variations between Huaye 1 and 93-11 reference genome.**

Concentric circles show the different features that were drawn using the Circos platform. The 12 chromosomes are presented along the perimeter of each circle. The circles from outside to inside indicate the coordinate of chromosome for SNP density, InDel density and SV density; the distribution unit of genome is 100Kb.

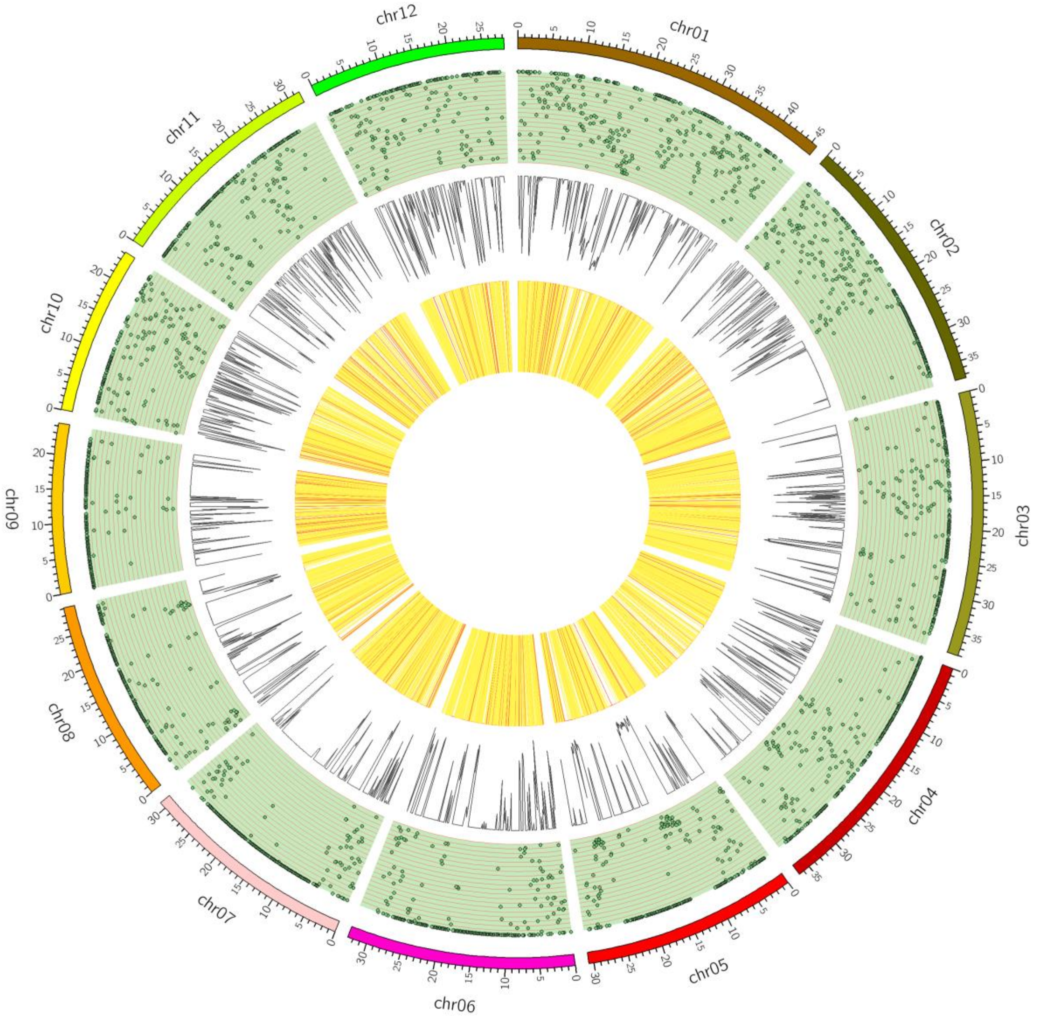


**Figure I. Summary of whole genome variations between Huaye 2 and Nipponbare reference genome.**

Concentric circles show the different features that were drawn using the Circos platform. The 12 chromosomes are presented along the perimeter of each circle. The circles from outside to inside indicate the coordinate of chromosome for SNP density, InDel density and SV density; the distribution unit of genome is 100Kb.

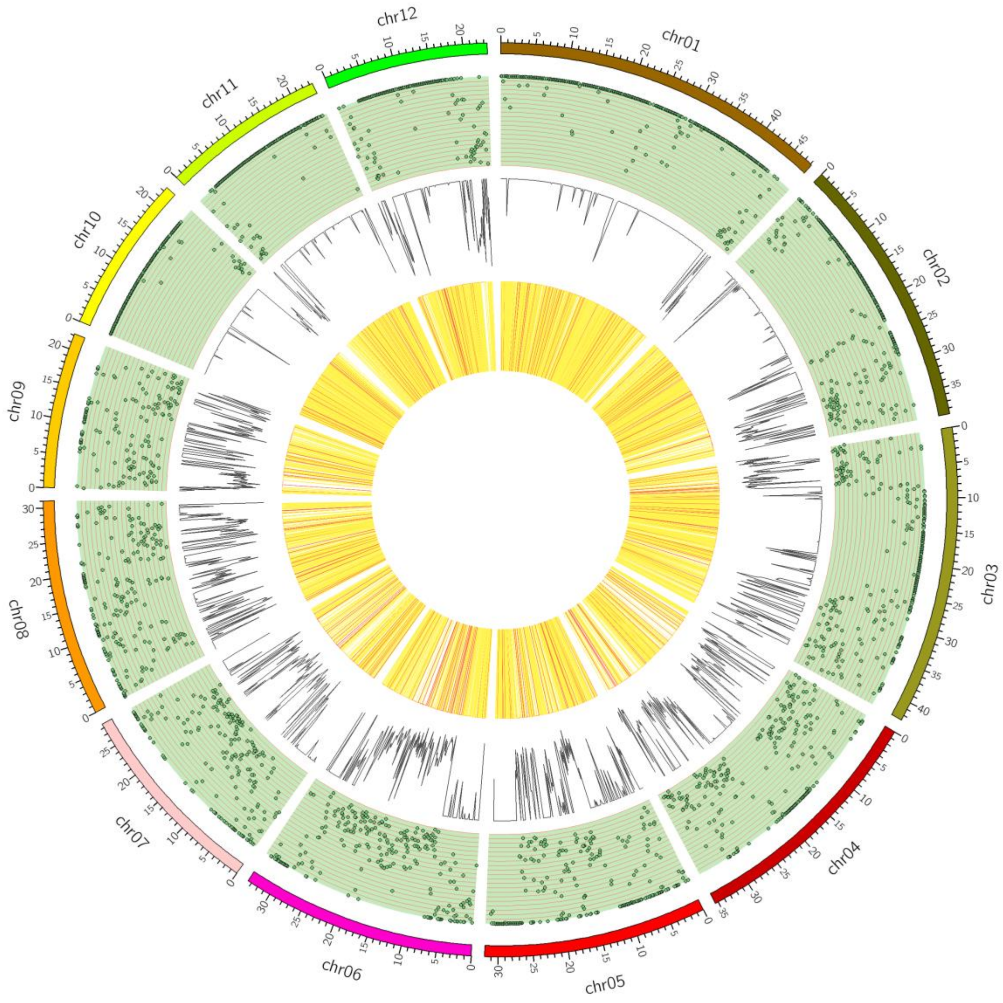


**Figure J. Summary of whole genome variations between Huaye and 93-11 reference genome.**

Concentric circles show the different features that were drawn using the Circos platform. The 12 chromosomes are presented along the perimeter of each circle. The circles from outside to inside indicate the coordinate of chromosome for SNP density, InDel density and SV density; the distribution unit of genome is 100Kb.

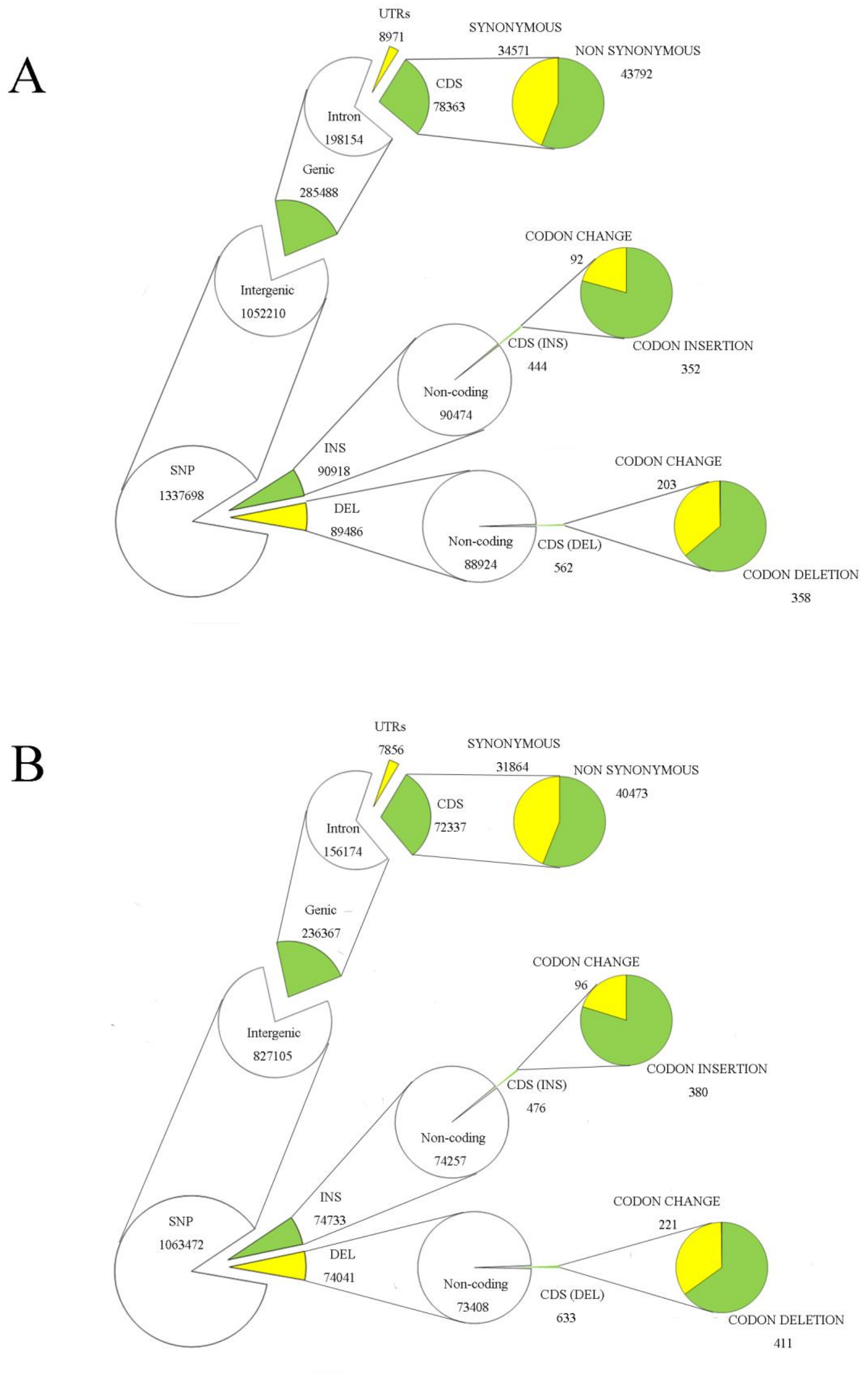


**Figure K. Annotation and distribution of SNPs and InDels between wild rice lines and 93-11 reference genome.**

SNPs, insertions and deletions on the rice pseudomolecules were classified as genic and intergenic, and locations within the gene models were annotated. The number of SNPs, insertions and deletions in each class is shown. A and B indicate the distributions of SNPs and InDels in the comparisons of Huaye 1 and 93-11 and Huaye 2 and 93-11 reference genome, respectively.

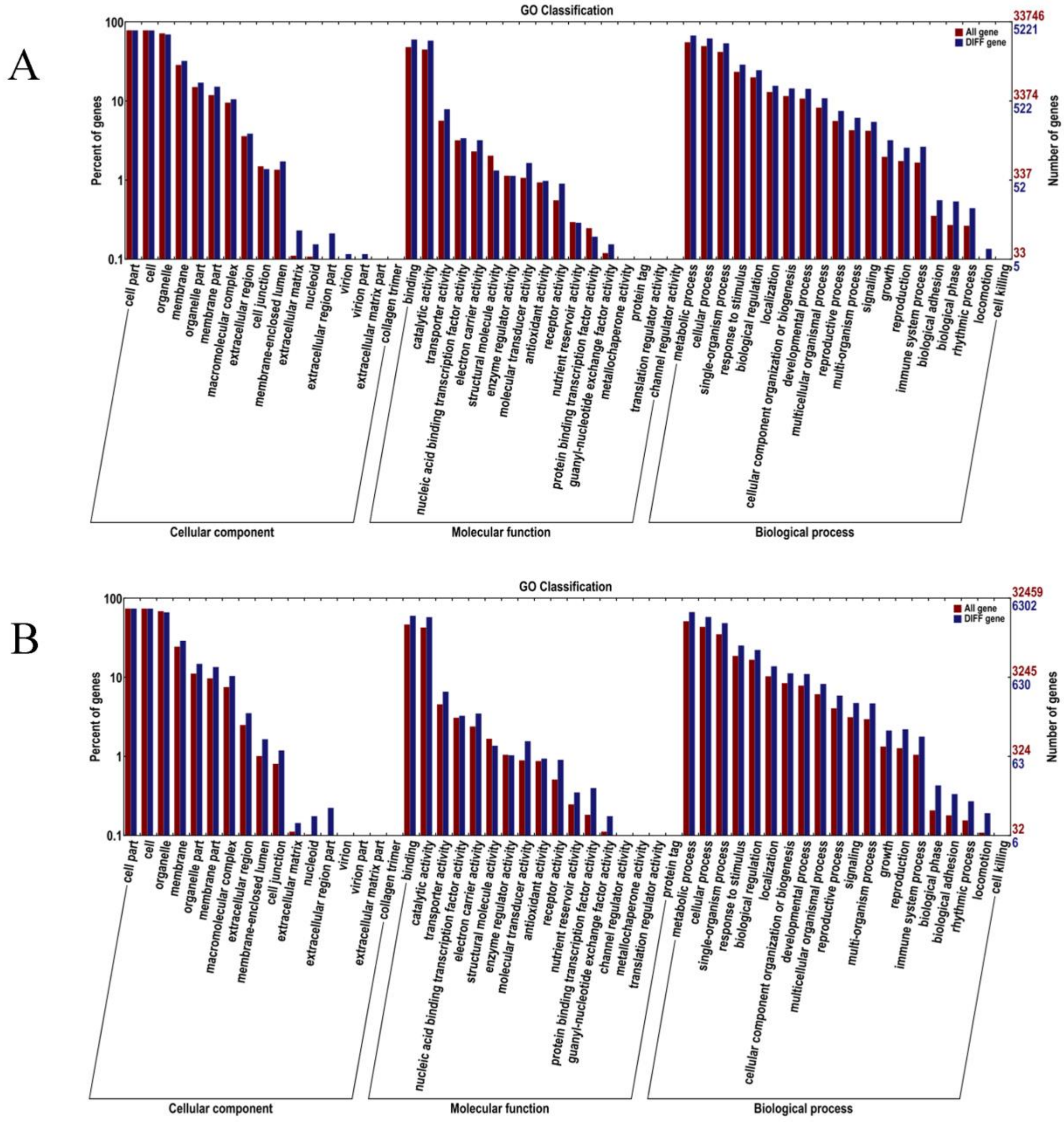


**Figure L. Gene ontology (GO) enrichment analysis of mutant genes detected in Huaye 1.**

(A) and (B) are showing GO analysis of mutant genes that were detected in the comparisons of Huaye 1 and Nipponbare, and Huaye 1 and Nipponbare reference genome, respectively. Abscissa is the GO classification, left ordinate indicates the percentage of genes, and right ordinate indicates the genes number and mutant genes number.

A

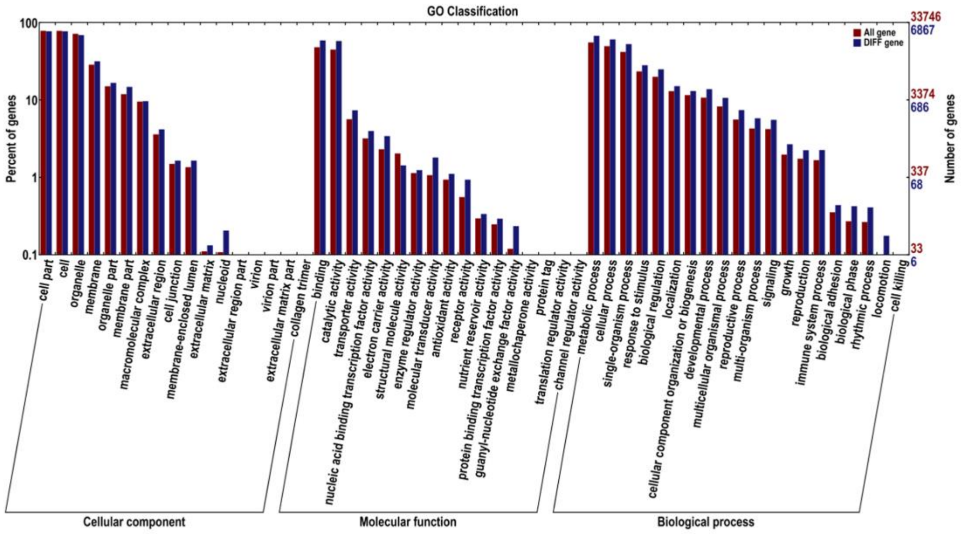


B

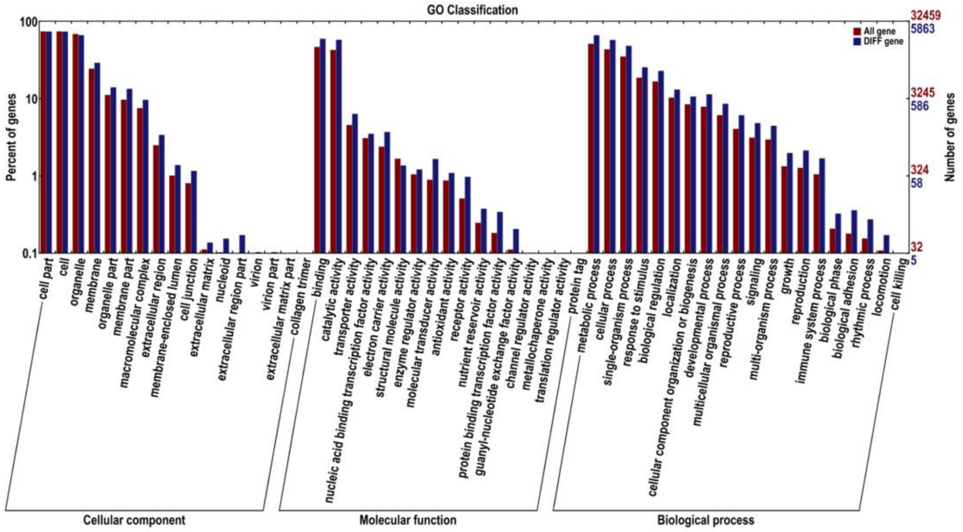


**Figure M. Gene ontology (GO) enrichment analysis of mutant genes detected in Huaye 2.**

(A) and (B) are showing GO analysis of mutant genes that were detected in the comparisons of Huaye 2 and 93-11, and Huaye 2 and 93-11reference genome, respectively. Abscissa is the GO classification, left ordinate indicates the percentage of genes, and right ordinate indicates the genes number and mutant genes number.

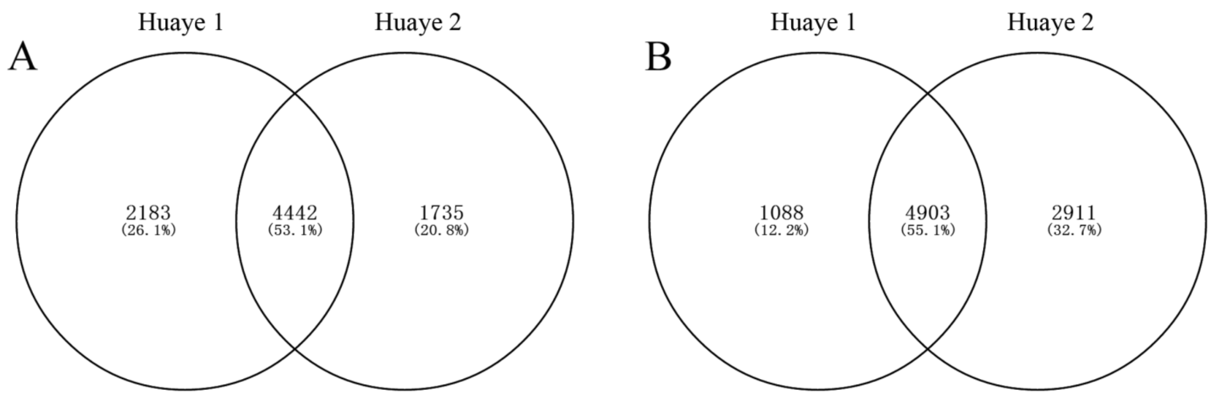


**Figure N. Venn diagram illustrating the proportion of shared gene clusters between two wild rice lines and reference genomes.**

(A) and (B) indicate the genes with sequence variations (SNPs and InDels) in wild rice lines compared to the reference genomes of 93-11 and Nipponbare, respectively.

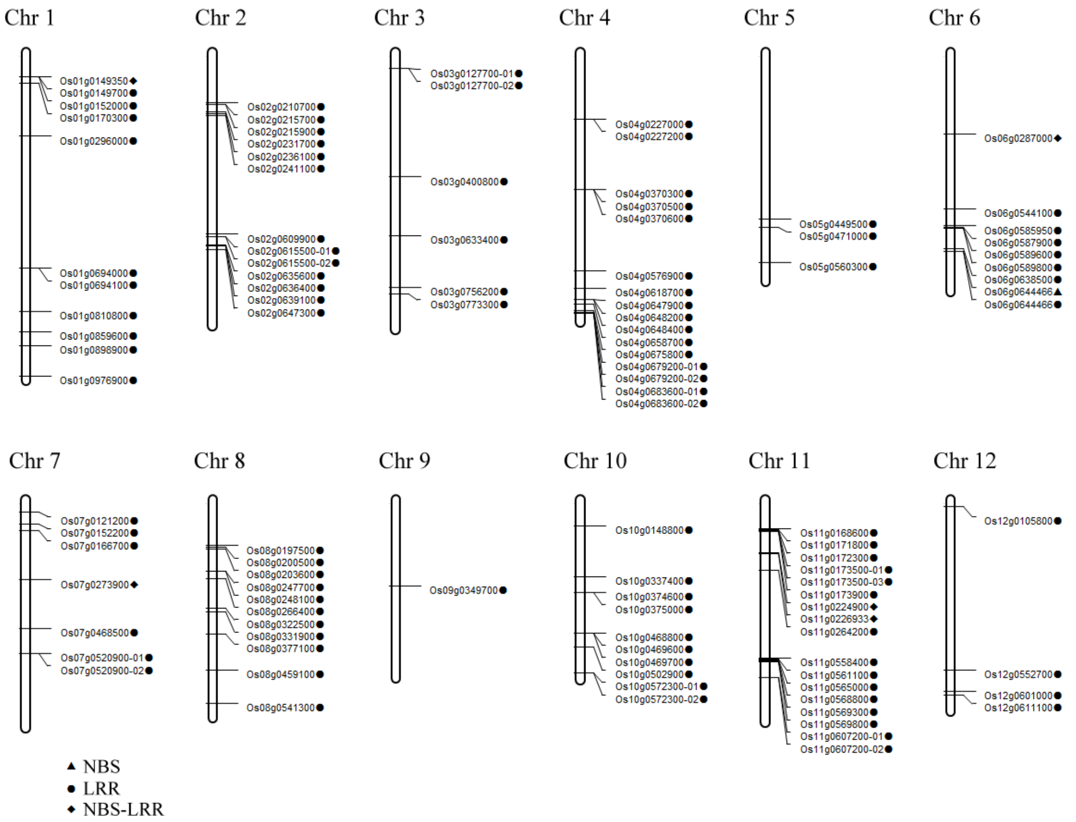


**Figure O. Distribution map of NBS-LRR resistance genes that were detected between Huaye 1 and Nipponbare reference genome.**

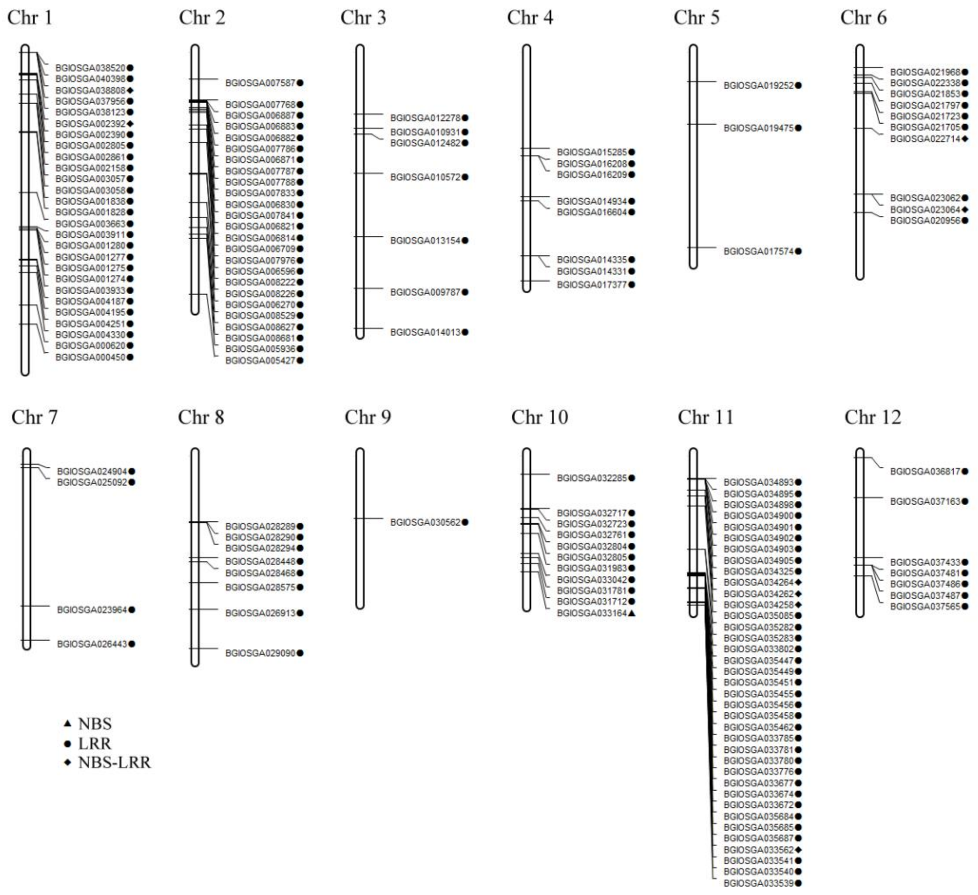


**Figure P. Distribution map of NBS-LRR resistance genes that were detected between Huaye 2 and 93-11reference genome.**

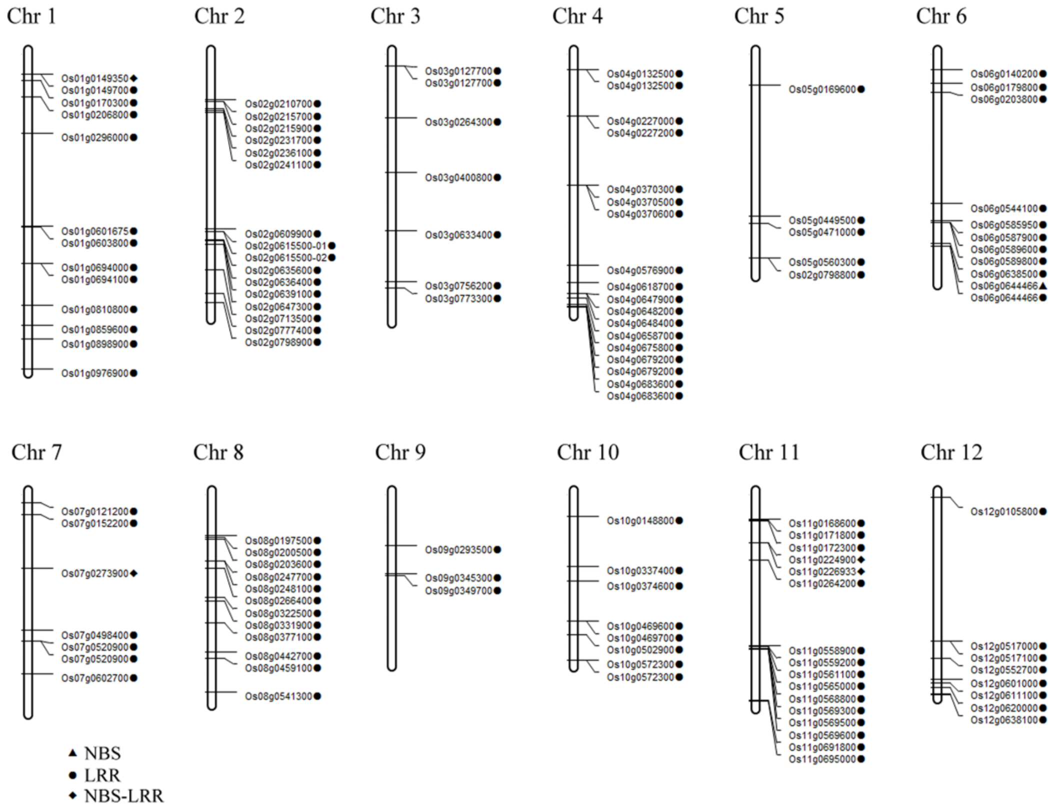


**Figure Q. Distribution map of NBS-LRR resistance genes that were detected between Huaye 2 and Nipponbare reference genome.**
