# Supplementary material for: Genome wide re-sequencing of newly developed Rice Lines from common wild rice (Oryza rufipogon Griff.) for the identification of NBS-LRR genes
Source: PLoS One. 2017 Jul 11;12(7):e0180662. doi: 10.1371/journal.pone.0180662 (PMC5507442; doi:10.1371/journal.pone.0180662)
Supplement: S1 Table — (DOCX) [file pone.0180662.s002.docx]

**S1 Table. Information of primers used for the verification of NBS-LRR genes.**

| Sample | genome | Gene ID | Product size (bp) | Forward primer sequence | Reverse primer sequence |
| --- | --- | --- | --- | --- | --- |
| Huaye 1 | 93-11 | BGIOSGA002391 | 696 | CTTTGATGGCAAGAGGAAGG | TGTTTGGCTTGGAGAGAACC |
|  |  | BGIOSGA002392 | 706 | AATATCACCTTGGGCACCTG | GCAACACCGTTTACCGAAAT |
|  |  | BGIOSGA022714 | 701 | CAGCTCGAAATGTGGATGAA | TGCAAGCACAGCTCCTATTG |
|  |  | BGIOSGA033164 | 699 | CCGATGTCTCTCGAAGGTTT | ATGAAGGCGACTACCCTGTG |
|  |  | BGIOSGA033536 | 225 | TCGCAACAACATCGTTCATT | GGCAGTGGGTTTCTCAATCT |
|  |  | BGIOSGA033562 | 701 | CAACCGTCGTCACACAAATC | AAGCGATGAAGAGGAAAGCA |
|  |  | BGIOSGA034258 | 695 | TGGTGTGTTGCTCTCTTTGC | GGATGGTCGTGTCTCAAACC |
|  |  | BGIOSGA034262 | 699 | CAAACGATGGCACCCTAAGT | GCCACAACGTCGATTTACCT |
|  |  | BGIOSGA034264 | 706 | CACCTCCTCCAGAGTGCTGT | GGGTTGCCAGCTCAAACTTA |
|  |  | BGIOSGA038808 | 694 | AGCTCAAGGGCGACAAGATA | TGTTTGGGAACCTGAACCAT |
| Huaye 1 | Nipponbare | Os01g0149350 | 710 | TTTCCAAACATCATCCAGCA | TCTTCAACGCTGTTCGTGAG |
|  |  | Os06g0287000 | 701 | CAGCTCGAAATGTGGATGAA | TGCAAGCACAGCTCCTATTG |
|  |  | Os06g0644466 | 701 | CAGAGCATCACAAAGCTGGA | TCTCCCAACCATTCTGGAAG |
|  |  | Os07g0273900 | 695 | ACAACGATGAGGCCAAGAAC | GTGGAAGAGGGCACCTACTG |
|  |  | Os11g0224900 | 698 | GATGCTGGGACTTGGTTGAT | AAGCTCGCCATTGAGATTGT |
|  |  | Os11g0226933 | 690 | GCTAAGTCCTTGCTGGCTTC | ACCAGAGGCAGTAGCTGAGG |
| Huaye 2 | 93-11 | BGIOSGA033164 | 699 | CCGATGTCTCTCGAAGGTTT | ATGAAGGCGACTACCCTGTG |
|  |  | BGIOSGA002392 | 706 | AATATCACCTTGGGCACCTG | GCAACACCGTTTACCGAAAT |
|  |  | BGIOSGA022714 | 701 | CAGCTCGAAATGTGGATGAA | TGCAAGCACAGCTCCTATTG |
|  |  | BGIOSGA023064 | 702 | GGCCACTAAGGTTTGTTTGG | TGATCCACCTAGCAGCACAG |
|  |  | BGIOSGA033562 | 701 | CAACCGTCGTCACACAAATC | AAGCGATGAAGAGGAAAGCA |
|  |  | BGIOSGA034258 | 695 | TGGTGTGTTGCTCTCTTTGC | GGATGGTCGTGTCTCAAACC |
|  |  | BGIOSGA034262 | 699 | CAAACGATGGCACCCTAAGT | GCCACAACGTCGATTTACCT |
|  |  | BGIOSGA034264 | 706 | CACCTCCTCCAGAGTGCTGT | GGGTTGCCAGCTCAAACTTA |
|  |  | BGIOSGA038808 | 694 | AGCTCAAGGGCGACAAGATA | TGTTTGGGAACCTGAACCAT |
| Huaye 2 | Nipponbare | Os06g0644466 | 701 | CAGAGCATCACAAAGCTGGA | TCTCCCAACCATTCTGGAAG |
|  |  | Os01g0149350 | 710 | TTTCCAAACATCATCCAGCA | TCTTCAACGCTGTTCGTGAG |
|  |  | Os07g0273900 | 695 | ACAACGATGAGGCCAAGAAC | GTGGAAGAGGGCACCTACTG |
|  |  | Os11g0224900 | 690 | GCTAAGTCCTTGCTGGCTTC | ACCAGAGGCAGTAGCTGAGG |
|  |  | Os11g0226933 | 690 | GCTAAGTCCTTGCTGGCTTC | ACCAGAGGCAGTAGCTGAGG |
