# Supplementary material for: Genome wide re-sequencing of newly developed Rice Lines from common wild rice (Oryza rufipogon Griff.) for the identification of NBS-LRR genes
Source: PLoS One. 2017 Jul 11;12(7):e0180662. doi: 10.1371/journal.pone.0180662 (PMC5507442; doi:10.1371/journal.pone.0180662)
Supplement: S2 Table — (DOCX) [file pone.0180662.s003.docx]

**S2 Table. BLAST validation of NBS-LRR gene sequences by NCBI**

| Sample | Genome | Gene ID | Ident (%)^*^ | Gene description in NCBI | Gene accession |
| --- | --- | --- | --- | --- | --- |
| Huaye 1 | 93-11 | BGIOSGA002391 | 100 | *Oryza sativa* Indica Group cultivar RP Bio-226 chromosome 1 sequence | CP012609.1 |
|  |  | BGIOSGA002392 | 99 | *Oryza sativa* Japonica Group NBSt1 gene for putative NBS-LRR disease resistance protein, complete cds | AB379818.1 |
|  |  | BGIOSGA022714 | 100 | *Oryza sativa* Japonica Group nitrate-induced NOI protein-like protein (Pi50_NIP) and NBS-LRR type R protein (Pi50_NBS1) genes, complete cds; and Pi50_NBS2 and Pi50_NBS3 pseudogenes, complete sequence | KP985759.1 |
|  |  | BGIOSGA033164 | 100 | *Oryza sativa* Indica Group NBS1-like protein mRNA, partial cds | FJ475131.1 |
|  |  | BGIOSGA033536 | 99 | *Oryza sativa* Japonica Group Pikm2-TS, Pikm1-TS genes for NBS-LRR class disease resistance proteins, complete cds, 198 Kb Tsuyuake BAC clone TS18H12 | AB462256.1 |
|  |  | BGIOSGA033562 | 99 | *Oryza sativa* (indica cultivar-group) NBS-LRR-like protein A (NL-A), NBS-LRR-like protein B (NL-B), NBS-LRR-like protein C (NL-C), and NBS-LRR-like protein D (NL-D) genes, complete cds | AY518220.1 |
|  |  | BGIOSGA034258 | 99 | *Oryza rufipogon* NBS-LRR-like protein NR59 gene, partial cds | AY169497.1 |
|  |  | BGIOSGA034262 | 93 | Oryza sativa Indica Group cultivar RP Bio-226 chromosome 6 sequence | CP012614.1 |
|  |  | BGIOSGA034264 | 100 | *Oryza sativa* Indica Group Os11gRGA3 pseudogene, NBS-LRR type protein, partial sequence, cultivar: Peh-kuh-tsao-tu | AB604620.1 |
|  |  | BGIOSGA038808 | 99 | *Oryza sativa* Indica Group cultivar Reiho NBS-LRR class disease resistance protein (Pi-km2) gene, complete cds | GU811865.1 |
| Huaye 1 | Nipponbare | Os01g0149350 | 97 | *Oryza rufipogon* cultivar Dal Dhan NBS-LRR disease resistance protein (Pit) gene, complete cds | KF741821.1 |
|  |  | Os06g0287000 | 100 | *Oryza sativa* Japonica Group NBS-LRR type protein (Pi50_NBS8), NBS-LRR type R protein (Pi50_NBS9), NBS-LRR type R protein (Pi50_NBS10), NBS-LRR type R protein (Pi50_NBS11), and NBS-LRR type R protein (Pi50_NBS12) pseudogenes, complete sequence | KP985760.1 |
|  |  | Os06g0644466 | 100 | *Oryza sativa* Japonica Group DNA, chromosome 6, cultivar: Nipponbare, complete sequence | AP014962.1 |
|  |  | Os07g0273900 | 100 | *Oryza sativa* Japonica Group genomic DNA, chromosome 7, BAC clone:OSJNBb0032G22 | AP005880.4 |
|  |  | Os11g0224900 | 100 | *Oryza sativa* Japonica Group Os11gRGA3 mRNA for NBS-LRR type protein, complete cds, cultivar: Nipponbare, Mokoto, Hitomebore | AB604618.1 |
|  |  | Os11g0226933 | 100 | *Oryza sativa* Japonica Group Os11gRGA8 mRNA for NBS-LRR type protein, partial cds, cultivar: Nipponbare, Mokoto, Hitomebore | AB604636.1 |
| Huaye 2 | 93-11 | BGIOSGA033164 | 100 | *Oryza sativa* Indica Group NBS1-like protein mRNA, partial cds | FJ475131.1 |
|  |  | BGIOSGA002392 | 99 | *Oryza sativa* Japonica Group NBSt1 gene for putative NBS-LRR disease resistance protein, complete cds | AB379818.1 |
|  |  | BGIOSGA022714 | 100 | *Oryza sativa* Japonica Group nitrate-induced NOI protein-like protein (Pi50_NIP) and NBS-LRR type R protein (Pi50_NBS1) genes, complete cds; and Pi50_NBS2 and Pi50_NBS3 pseudogenes, complete sequence | KP985759.1 |
|  |  | BGIOSGA023064 | 96 | *Oryza sativa* (indica cultivar-group) NBS-LRR-like protein A (NL-A), NBS-LRR-like protein B (NL-B), NBS-LRR-like protein C (NL-C), and NBS-LRR-like protein D (NL-D) genes, complete cds | AY518220.1 |
|  |  | BGIOSGA033562 | 99 | *Oryza sativa* (indica cultivar-group) NBS-LRR-like protein A (NL-A), NBS-LRR-like protein B (NL-B), NBS-LRR-like protein C (NL-C), and NBS-LRR-like protein D (NL-D) genes, complete cds | AY518220.1 |
|  |  | BGIOSGA034258 | 99 | *Oryza rufipogon* NBS-LRR-like protein NR59 gene, partial cds | AY169497.1 |
|  |  | BGIOSGA034262 | 93 | *Oryza sativa* Indica Group cultivar RP Bio-226 chromosome 6 sequence | CP012614.1 |
|  |  | BGIOSGA034264 | 100 | *Oryza sativa* Indica Group Os11gRGA3 pseudogene, NBS-LRR type protein, partial sequence, cultivar: Peh-kuh-tsao-tu | AB604620.1 |
|  |  | BGIOSGA038808 | 99 | *Oryza sativa* Indica Group cultivar Reiho NBS-LRR class disease resistance protein (Pi-km2) gene, complete cds | GU811865.1 |
| Huaye 2 | Nipponbare | Os06g0644466 | 99 | *Oryza sativa* Indica Group cultivar RP Bio-226 chromosome 6 sequence | CP012614.1 |
|  |  | Os01g0149350 | 97 | *Oryza rufipogon* cultivar Dal Dhan NBS-LRR disease resistance protein (Pit) gene, complete cds | KF741821.1 |
|  |  | Os07g0273900 | 100 | *Oryza sativa* Japonica Group genomic DNA, chromosome 7, BAC clone:OSJNBb0032G22 | AP005880.4 |
|  |  | Os11g0224900 | 100 | *Oryza sativa* Japonica Group Os11gRGA3 mRNA for NBS-LRR type protein, complete cds, cultivar: Nipponbare, Mokoto, Hitomebore | AB604618.1 |
|  |  | Os11g0226933 | 100 | *Oryza sativa* Japonica Group Os11gRGA8 mRNA for NBS-LRR type protein, partial cds, cultivar: Nipponbare, Mokoto, Hitomebore | AB604636.1 |

*Sequence identity (%)
