# Supplementary material for: Genome wide re-sequencing of newly developed Rice Lines from common wild rice (Oryza rufipogon Griff.) for the identification of NBS-LRR genes
Source: PLoS One. 2017 Jul 11;12(7):e0180662. doi: 10.1371/journal.pone.0180662 (PMC5507442; doi:10.1371/journal.pone.0180662)
Supplement: S3 Table — (DOCX) [file pone.0180662.s004.docx]

**S3 Table. The distribution of NBS-LRR genes in rice genome.**

| Chromosome | Huaye 1 vs 93-11 | |  | Huaye 1 vs Nipponbare | |  | Huaye 2 vs 93-11 | |  | Huaye 2 vs Nipponbare | |
| --- | --- | --- | --- | --- | --- | --- | --- | --- | --- | --- | --- |
|  | Genes | Gene cluster |  | Genes | Gene cluster |  | Genes | Gene cluster |  | Genes | Gene cluster |
| 1 | 19 | 5 |  | 11 | 2 |  | 27 | 6 |  | 13 | 3 |
| 2 | 24 | 3 |  | 13 | 3 |  | 25 | 3 |  | 16 | 3 |
| 3 | 5 | 0 |  | 6 | 1 |  | 7 | 0 |  | 7 | 1 |
| 4 | 11 | 1 |  | 16 | 5 |  | 8 | 2 |  | 18 | 6 |
| 5 | 2 | 0 |  | 3 | 0 |  | 3 | 0 |  | 5 | 1 |
| 6 | 9 | 1 |  | 9 | 2 |  | 10 | 1 |  | 11 | 2 |
| 7 | 7 | 0 |  | 7 | 0 |  | 4 | 0 |  | 7 | 1 |
| 8 | 9 | 1 |  | 11 | 2 |  | 8 | 1 |  | 12 | 2 |
| 9 | 7 | 2 |  | 1 | 0 |  | 1 | 0 |  | 3 | 0 |
| 10 | 9 | 1 |  | 10 | 3 |  | 11 | 2 |  | 8 | 2 |
| 11 | 33 | 6 |  | 17 | 4 |  | 37 | 7 |  | 16 | 4 |
| 12 | 8 | 1 |  | 4 | 0 |  | 7 | 1 |  | 8 | 2 |
| Total | 143 | 21 |  | 108 | 22 |  | 148 | 23 |  | 124 | 27 |
